# Supplementary material for: Active inference and artificial reasoning
Source: Nat Commun. 2026 Sep 8;17:10125. doi: 10.1038/s41467-026-77209-5 (PMC13601584; doi:10.1038/s41467-026-77209-5)
Supplement: Supplementary file 1 [file 41467_2026_77209_MOESM1_ESM.pdf]

nature portfolio

Peer Review File

Active inference and artificial reasoning

Corresponding Author: Dr Thomas Parr

This file contains all reviewer reports in order by version, followed by all author rebuttals in order by version.

Attachments originally included by the reviewers as part of their assessment can be found at the end of this file.

Version 0:

Reviewer comments:

Reviewer #1

(Remarks to the Author)
This paper is a mathematically interesting contribution that extends expected free energy with an explicit information gain over models, alongside the usual gains over states and parameters. That is a genuine strength: the authors do not merely argue that reasoning can be cast as model selection, but provide a concrete variational framework that integrates model comparison into active inference. A second strength is the clean organization across timescales, separating inference, learning, and model selection in a way that is conceptually elegant and internally consistent.

The practical side is also handled well within the chosen setting. The three-ball task is simple, but it is genuinely partially observed, so the agent must decide what to inspect before it can answer correctly. This makes the example more meaningful than a standard fully observed toy problem. The ablation results are another clear strength: they show that information gain over models is not just a theoretical addition, but materially improves discovery speed and overall task performance.

My main mathematical concern is that the method still depends heavily on a hand-crafted hypothesis space. The variational derivations are elegant, but much of the real difficulty is pushed into specifying the right set of candidate models in advance. In that sense, the framework is strongest once the correct model class is already available. I also find some design choices insufficiently principled, such as the thresholding and count-scaling heuristics, which seem more pragmatic than theoretically grounded. WOuld it be possible to assume less prior knowledge about the models? What prior knowledge would be plausible from a neuroscience perspective? I encourage the authors to consider those two questions.

My main practical concern is scalability. The paper demonstrates the approach on a carefully structured toy problem where the representation is largely known and only one key mapping must be inferred. That is a useful proof of concept, but it falls short of showing practical reasoning in more realistic settings with richer observations and much larger hypothesis spaces. Overall, I find the paper strong in mathematical formulation and clear in its practical motivation, but still limited by its reliance on manual model design and its narrow empirical scope. If the hypothesis space is much larger, could one rely on naive mean-field approximations in variational inference to keep the computations tractable? I recommend the authors to address the scalability issue.

(Remarks on code availability)

Reviewer #2

(Remarks to the Author)
Review: Active inference and artificial reasoning
In this article, the authors propose an efficient method for extending the expected information gain component of the expected free energy, used as objective function for selecting action ins active inference, so that is also includes the expected information gain about model structure. They show in simulation studies that this method, which applies specifically when using Bayesian model reduction under fully discrete partially observed Markov decision process generative models, leads to increased sample efficiency as compared to only using information gain about states and parameters. The paper is well-written and consistent, and the introduced methodology is certainly relevant for the use of active inference models in the various contexts where they are used, including in artificial intelligence and for computational

phenotyping such as in computational psychiatry. I am therefore happy to recommend this work for publication. I do, however, have a set of minor recommendations that I hope can contribute to improving the manuscript, such as regarding clarity, discussion of limitations, situating the work in the broader literature, and regarding the simulation methodology. Unless otherwise is mentioned, these should be seen as optional.

Clarity: technical extension or model of reasoning.
Throughout the article, it can feel, to me, somewhat unclear whether the aim of the work is to technically extend the active inference framework, or to provide a computational implementation of a theory of (human) reasoning. This should be made clear from the beginning.
If the aim is indeed to provide a model of reasoning, the paper should also include at least a brief overview of the literature already engaging with this goal, beyond the (often somewhat casual) use of the term in language models. It should also be justified why, exactly, being sensitive to information gain in model space when choosing where to look constitutes a model of reasoning. Finally, it would be useful to have at least a brief sketch in the discussion of how one might test this account of reasoning empirically in the future, such as by collecting behavioural data from reasoning tasks and investigating whether the augmented information gain term improves the ability of the model to capture the behaviour.
If the purpose is rather to technically extend the active inference framework to be sensitive to information gain in model space, and the term “reasoning” is used more casually here, the authors might make this more explicit early in the article.

Clarity: technical terminology
The article (and the active inference framework) uses a quite extensive technical terminology that is quite specific to its field, but also sometimes varies between articles and authors in it. It would be quite helpful to have an overview of this early on, perhaps in a small box. Here I mean terms like “state”, “factor”, “observation”, “modality”, “path”, “policy”, “action”, “parameter”, “belief” and so on. This is especially relevant because different terms are used to describe the dimensionalities of states, observations and actions (i.e., factor, modality, and I am unsure what the value of a single timepoint for a single dimension of an action is called). This could also include the mathematical annotation for the different terms. Establishing such an overview might be nice for the active inference field in general.
The authors use “path” to refer to a single dimension of the action over time (distinguishing it from a “policy”, which refers to all the different action dimensions). In other fields, “paths” would not necessarily be considered one-dimensional, nor would they only refer to actions. Perhaps there is a better term than “path”? In any case, it is not clearly introduced before it is used in the figure text for Figure 1, which I expect would be confusing to many readers.
The authors early on use the term “learning” to refer specifically to parameter learning, and distinguish it from “selection”, which refers to model selection. However, they also call the model selection “rule learning”, and mention “learning a generative model”. To be consistent in their terminology, they might change “learning” to “parameter learning”, and use “learning” for parameter and model learning in general; or they might not use the term “learning” for the Bayesian model selection, despite the fact that this is often called “structure learning” in other fields. Finally, if the authors here intend for “structure learning”, “rule learning” and “learning a model” to be synonymous, they might make this explicit; if not, they might clarify the relations between these terms.
The authors call the kind of Bayesian model selection used in the paper a post-hoc approach. They might early on make clearly exactly what is meant with this. What makes it post-hoc?

Clarity: formal details
It might be useful if the digamma function and its role in the formalism is introduced briefly, to users who are not familiar with it.
If I understand correctly, a model-average of the various possible models is used in the beginning, until a single model is committed to. During this pre-commitment phase, parameter learning is done across all the possible models, which is feasible because it still only has to be done once (under this model reduction scheme) – the parameters can be reused, so to speak, because the different models are versions of the full model and the updated parameters are the same across models. Is that correct? Or does parameter learning not begin until the model has been selected?
Since I was a bit in doubt, perhaps it is useful to clarify this in the article. Finally, it might be discussed whether there are formal limitations to this approach to Bayesian model reduction, at least if my first interpretation is correct. Is it an approximation, or would it give the same results as if one used the (computationally heavy) approach of calculating the variational free energy for each sub-model separately and compared their model evidences?

Clarity: structure of introduction
The section titled “Active Inference, Learning and Selection” has some overlap with the introduction, and some of it would make reading the introduction easier. Section such as p. 3 starting with “Technically, variational free energy minus” would perhaps fit better in the “Active Inference, learning and selection” section, for example. The authors might consider if there is a way to streamline this first part of the article more.

Clarity: overview of contributions
The paper seems to make several, distinct, and increasingly specific, contributions. One is treating the choice of model structure as a random variable in its own right, and including the expected information gain for this variable in the expected free energy. Another is to apply this specifically for the already introduced case of Bayesian model reduction used here, where submodels of the full model can be specified by setting parameter values to 0. A third is to provide a manageable subset of models for the three-ball task (by imposing various sparsity constraints) which allows using the above method in practice. A final contribution is to provide an Ockam’s Razor approach where the agent moves from having a belief distribution about which model is true to committing to using only one model, only after which it has preferences about its observations. Giving a clear overview in this fashion early in the article might be useful for readers to understand where the contributions are, and how they depend on each other.

Limitations: specific generative model type
The authors use a somewhat specific type of generative model here, namely a discrete state-, observation-, action-, time- and model-, but continuous parameter-, space partially observable Markov decision process. Only laten states have dynamics and depend on their own previous state; observations only depend on states, and parameters have no dynamics at all. In addition, it is only transition models parameterised by B that depend on actions (and not the observation model parameterised by A, nor the model space). This model family is of course ubiquitous in the active inference as generative model, but it might still be useful to clarify briefly the limiting assumptions it makes in general. More specifically, it would be useful to discuss whether the methods introduced in this article also apply to other types of generative model, such as continuous state space models.
It might also be useful to clarify to which degree further limiting assumptions are imposed by this type of Bayesian model reduction and calculation of the model space expected information gain. In its current form, it seems to be incompatible with temporally deep planning. Is it also incompatible with hierarchically deep generative models? It does seem compatible with counterfactually deep generative models. Is it compatible with sophisticated inference or inductive inference? Would it interact in any relevant way with either of the above?

Limitations: only compatible with Bayesian model reduction
The authors might point out that the method they use is here is compatible only for use with the type of Bayesian model reduction they use (although not the general principle or including information gain about a random variable controlling the choice of model). This of course has advantages, as the authors mention, such as making the calculation highly efficient. First, the authors might motivate this requirement for speed a little; is it computationally infeasible to use other methods, or is the speed primarily relevant for applications that are highly speed-sensitive?
In addition, the authors might consider to which degree this Bayesian model reduction approach is plausible as a model of (human) cognition. Is it likely that humans would separately evaluate a model evidence for a pre-specified set of submodels? 81 models still seem many, at least for it to be done consciously. Additionally, this method require the model space to be fully pre-specified. In which cases could this be expected?

Limitations: rule learning, not structure learning
In this article, the authors focus specifically on rule-learning, which is arguably a special case of the much broader task of structure learning. In this context, the authors use a series of constraints to a priori reduce the space of possible models. This includes (if I understand correctly) assuming that a) a set of latent states only can result in a single feedback outcome, b) that rules are always a combination of an action and a single criterion latent state, and c) that a criterion laten state is fully determined by a single latent context state. These assumptions are of course appropriate for this version of the three-ball paradigm, but do not apply even to slightly different versions of the task (such as if the correct answer depended on more than a single position). The authors should make these limitations explicit early on (they are stated, but are somewhat difficult to read). A visual depiction of how these constraints limit the space of possible models would be quite useful, I think. The authors might discuss to which degree their approach might generalise to other cases, and discuss to which degree this furnishes a generally applicable way to a priori limit the model space. Finally, they might discuss whether a human participant is likely to approach this task with the same constraints or not.

Limitations: the three-ball paradigm
The authors might discuss the limitations imposed by using the three-ball paradigm in their simulations. It is for example perhaps a task where the information in model space is quite strongly dependent on the set of cues given to the agent, and arguably less dependent on the action the agent takes – in other words, I wonder if it is a task where the effect of including model space expected information gain is smaller than it might be in a task where action very strongly determine the effect of information one gets about model outcomes. The three-ball paradigm is also a task where, it seems, a main avenue for model space information is to make guesses given the cues a lot (as is seen in the simulation), which quite starkly impacts the pragmatic gain under non-flat preference priors (probably why flattening the preference priors is necessary in the beginning). One could easily imagine other tasks where it is possible to have preferences also during the model selection process.

Limitations: Occam's Razor to one model
The authors use what they call an Ockam's Razor approach, where the agent has an initial phase where it does Bayesian model comparison, until the Bayes factor reaches a threshold that triggers committing fully to a single model. Only after this does the agent have preferences for the observations it gets.
The authors should discuss this modelling in more detail, and discuss the alternatives.
First, what are alternatives? Arguably, a simpler approach is to just to use a Bayesian model average of the different models when planning, for example. If the posterior in model space is sharp, it seems this should become identical to the single-model approach.
Second, what are the justifications for the approach in the paper? Is it primarily the lessened computational load of committing to a single model? Is it informational, that averaging across models will lead to unidentifiability in state inference, for example? Or is it motivated by an account of (human) cognition, where people necessarily commit to a single hypothesis before acting on it? Or is it rooted in other principles from the Bayesian mechanics that active inference rely on ?
Third, what are the limitations of the method? It is for example not possible to change one's mind after committing to the model, even if strong evidence against it is provided afterwards.
Fourth, the authors might discuss the fact that they use a threshold for choosing the model (as opposed to a continuous belief change, which is often presented as an advantage of Bayesian methods over other approaches). The choice of threshold seems somewhat arbitrary; would the authors expect to later let this threshold depend on a component of the generative model(s), such as a cost of being wrong in model space? Is there a way to derive this threshold in a principled

manner? Would the threshold be context-sensitive?
Finally, the authors might discuss and justify the choice of letting the agent have flat prior preferences before committing to a model.

Background: other kinds of structure learning
The Bayesian model reduction scheme used here is of course a special case of Bayesian model selection and structure learning in general. It could be useful if the authors situated their work somewhat within the larger field of Bayesian model selection, especially to discuss to which degree expected information gain could be calculated within other approaches. Would this approach for example be useful in the context of causal discovery, as a way to select informative actions about causal connections in a generative model (such as in Dynamical Causal Modelling)? Could it be used for model comparison where it is not the connections but different dynamics that are compared instead (such as is often done when modelling continuous state space phenomena). Is there a reason why priors in model space must be flat, beyond the use for the specific task here where we assume the agent has no previous knowledge about rules? Could it be combined with traditional methods that use (approximations of) the model evidence to select between models? Since a random variable controls the model, could this random variable have dynamics (i.e., a game where the rule changes, so that a different model becomes true at some point during the game)?
An obvious extension, which may seem closer to (human) reasoning, and which could benefit greatly from the sample-efficient information seeking behaviour provided by this article, would be in open-ended structure learning scenarios where a model is "grown" (i.e., where new causes are added and removed iteratively), such as in Dirichlet process models. The authors might briefly discuss whether and to which degree it would be possible to extend their method to include these use cases in the future.
Finally, there is also other work where Bayesian model selection is implemented by having a random variable control the model, such as in hierarchical mixture models. I know these best from fields using other approximations, such as sampling, to Bayesian inference, but they might still be worth mentioning in this context. Finally, the authors might discuss whether they would, in general, consider Bayesian model selection a special case of Bayesian inference over a latent variable that select between different model structures, or if this is a specific approach used in this article that should be considered distinct from other kinds of Bayesian model comparison.

Simulation methodology:
The authors run several simulation studies to investigate the effect of adding the model space expected information gain to the expected free energy. They do not clearly show this effect, however, since they show the difference when removing expected information in both model and parameter space simultaneously. Since this is arguably the main result in the article, it should be shown clearly. Secondly, they run the simulation only using the same initial conditions, and with the same set of cues, which makes it difficult to know how well their results generalise. I would also strongly recommend addressing this. See the last section with the specific comments for more information.

Other suggestions for discussion
The authors might consider briefly sketching out how the current formulation would interact with postdiction (i.e., re-inferring past states when receiving new information). As I understand, it is already implicit in the message passing scheme used that beliefs about a given state depend on knowledge about later states. Information gain in model space would supposedly also help disambiguating beliefs about past states.
The authors might discuss briefly whether this augmented version of the expected free energy is also derivable from the free energy principle. I expect it to be, since it seems to be just treating the model as another random variable to have beliefs about.
The authors might discuss how this kind of model reduction might be implemented neuronally; would it be a sudden change in connection strengths between neurons, or some other mechanism? How does it relate to proposed neurobiological implementations of the Dirichlet parameters, which the model reduction seems to act on?

Language, formatting and minor suggestions
p.1 "exploratory, information-seeking actions": one could also add that "exploration" terms in many models are also essentially just added randomness or propensity to take actions not taken before, where it is here targeted specifically at sampling observations that are expected to be informative given current beliefs.
p.1 "homologue of "thinking": the authors might justify this briefly, or at least indicate that it depends on what one means with "thinking". Indeed, thinking is sometimes defined as language-based or explicit/aware, neither of which are a priori compatible with the planning as inference account.
p.2 "one cannot learn to be curious": I would expect one could train a mapping from current beliefs and observations to the expected free energy – perhaps I am mistaken
p.2 "The So,": typo
p.3 "cover abstraction": the authors might clarify how exactly abstraction and reasoning are equated, and also why model space information gain sensitivity could be considered a type of abstraction.
p.3 "under the partially observed": the authors might here and henceforth make clear that they refer specifically to discrete state, action and observation POMDPs, since the following formalism doesn't seem to apply to continuous formulations. They do mention this at times, but it might be nice to clear from the beginning.
Figure 1. "empirical prior": This use of the term seems to diverge from how it is used in Bayesian inference to describe priors chosen based on previous work and data. Since "empirical" seems to denote something dependent on empirical data, it seems somewhat odd to use it for priors that depend on other variables in the model. Perhaps there is a standard term for this in the broader Bayesian inference world? I suppose this is often what the term "hyperprior" targets, although this of course describe the random variables which the "empirical priors" depend on.
Figure 1. The optimisations in the bottom-right corner of the figure are not explained in much detail in the figure text, and only

some of it in the main text quite a bit later (including the cursive l function of the E matrix, or the difference between the bold and non-bold matrix names). Perhaps giving a brief explanation here would be useful.
Equation 1: Should there be a subscript T for the variational free energy F on the left side? I suppose this will be the F at a specific point in time, and the right hand side of the equations uses t subscripts.
Equation 1: the dependence on the model m is explicated for the log model evidence p(o|m). I suppose the dependence on the model is also implicit in many of the other terms, however. The authors might consider making this consistent, and if not, stating clearly that omit the model dependency only in some terms. This is particularly relevant because this paper focuses specifically on model structure learning.
Equation 1: when calculating the prior in the first line, the transition from s-1 to 1 is calculated relative to the action u at time t (not t-1). Perhaps I am mistaken, but I would expect that it is the action at the previous timestep that controls this transition, since the action at the current timestep hasn't happened yet.
Equation 1: In the final line, in the term for calculating the complexity, I suppose the true posterior over states, actions and parameters will also be conditional on the previous action, and not just the previous state?
Equation 2: The authors point out that they omit some parts of the full equation here. This is fine, but of course can make the mathematics more opaque. I would find it useful if they would include a fully spelled out version of this and other equations in the appendix.
Equation 2: In the second equality, expected information gain formulations are used, and in the third equality, the risk and ambiguity formulation is used instead. However, the expected information gain about parameters seems to stay in the same form (except formulated as a KL divergence). Does the beliefs over parameters not have an ambiguity formulation, for example?
p.8: It might perhaps be useful to distinguish the predictive posterior from the posterior over states notationally somehow, such as with a tilde above the q, or similar.
p.8. "Here, Q:= P…". a seems to be used instead of A here.
p.8. "The prior in (3)…" equation 3 seems to have multiple priors – perhaps it can be explicated which one is meant.
Equation 5: mu is here used as a function of the Dirichlet parameters a and b. I presume this means taking the mean of the Dirichlet distribution to get the categorical distribution. If so, this might be made explicit.
Equation 5: The authors might make clear that these equations are now implementations of the previous equations, and are specific to the discrete POMDP generative model they use in the paper, whereas the previous equations were more general.
Equation 5: this equation might be explained in a little more detail, similar to how the previous equations were explained.
Equation 7: While this equation is probably described and motivated in more detail in the article referred to, it might be nice to go a bit more in depth here, since it is central to the extension this paper presents. This includes making clear what the stylised B represents, and why the final equality is the case.
p. 12 "entail a high mutual information". Why is it the case that there is a high mutual information between latent states and observations in the full set of models? Does the formalism not hold if some of the models being considered imply a low mutual information?
Equation 10: why are m and A not used in the parameter and state information gain terms here? Are they marginalised, omitted for simplicity, or is it a consequence of the factorisation into model, parameter and state space?
Equation 11: the delta used as left hand side in the final equality is not explained anywhere; it would be useful to do so, I think.
p. 13 "note that this expected…" Why does the expected information gain not work unless parameters are updated over successive time points? And why are they not? Is this by the assumption of separated timescales, where beliefs over parameters are not updated on every time point, or for a different reason?
p. 15. "preclude a learnable state-action policy" Why exactly is it not possible to use traditional reinforcement learning methods for sequential policy optimisation (or for partially observable environments)? And is this also the case when using standard Bayesian learning methods, such as the standard parameter learning scheme in active inference discrete POMDPs?
p. 16. "selected at random". Does this mean that the colour of the ball (i.e., the environment) depends on the agent's prior? Or does it refer to the agent assuming that the ball is sampled from D? And similarly, is there a difference between having a prior over the initial state (which may be fixed), and assuming that the initial state is sampled randomly? Here the second sounds like it is the case, but I would usually think of priors over latent states in the former way myself, so some clarity might be useful.
p. 16. The active inference model here selects its two actions (where to look and which colour to choose as its response) simultaneously. If a human were to do the three-ball task in an experimental setting, they would probably do them one at a time (i.e., first look, then decide, then look and so on). The authors might briefly comment on this, if nothing else just to make sure that the reader is not confused by expecting it to happen one at a time.
p. 18. "Avogadro's number": the authors might briefly explain why this is relevant here
p. 18. "a true outcome": this is ambiguous – maybe write that they generate an outcome that the response was correct
p. 21. "pose or colour": typo
Figure 4: the lowest two panels to the left have "trials" on the y-axis, while it is on the x-axis on all other panels in this figure. I would suggest changing this to keep it consistent and easier to read.
Figure 4: the third panel, titled "divergence" seems to not be explained in the figure text. The divergence between what? It seems to be between the true and estimated parameter values.
There are often a lot of repetition between figure texts and the main text (such as for Figure 4). The authors can consider if this is necessary, or if some of the figure texts could be removed, in particular the parts that pertain more to the interpretation of the figures.
Figure 6: Would it be useful to do this analysis only with information gain about models, or only about models and parameters, or is there are principled reason to only use the four settings in the current plot?
Figure 6: It might be nice to get a sense of the resulting parameter estimates (since these are arguably the point of the model reduction): do they get close to the true parameter values?

p. 26. “using the same initial conditions for each trial”: Why repeat the analysis with the same initial conditions? It would be nice to see if the simulations are robust to different initial conditions or not. It seems the plots in figure 7 could also easily accommodate more data points.
p. 27. “discovery time from”: typo
Figure 7: since it is the primary focus on the article, there should arguably be a panel showing the effect of just removing expected information gain about models, without also removing the expected information gain about parameters. Otherwise, it is hard to judge whether the additional information gain indeed does have an advantage in this specific task.
Figure 7: “same sequence of cues”. Does this just mean that there are the same ball colours on each trial across simulations? Is this the same as having the same initial conditions? At first I thought the cues would be action-dependent, since they depend on where the agent looks – in this case, having the same cues across simulations would not be appropriate. This might be clarified by the authors, and, as I said, they might extend the simulation to different sets of cues to see how well their results generalise.
p. 28. “Here, partial observability…” Arguably, the most relevant unobservable variable here is the rule / criterion, rather than the color of the other balls.
p. 29. First paragraph: this description of the Bayesian model reduction approach would perhaps be nice to have earlier in the article, when the method is introduced.
p. 29. “’what’ and ‘where”: it seems here that the above formulation requires a factorisation of latent states into these two classes, but it is unclear whether this is a formal necessity for the kind of inference shown in this paper, or derived from the general statement that (human) brain have this factorisation. It seems to me that the model reduction and the calculation of the model space expected information gain do not specifically require the “what” and “where” factorisation.

(Remarks on code availability)
I have not reviewed the code underlying the simulation. It is included in the most recent release of the SPM software, although the authors have not specified where the file is or what it is called. I am happy to review the code, but would need to know which file refers to the simulations used in this article.

Reviewer #3

(Remarks to the Author)
The authors extend the active inference framework beyond state and parameter inference to structure learning via Bayesian model reduction, generalising the expected free energy objective over a predefined model space. An Occam's razor criterion applied to model posteriors yields a formal account of curiosity-driven inference and moments of realisation. The approach is validated on the three-ball paradigm, a rule-based visual search task, with empirical focus restricted to likelihood mapping discovery within a carefully constrained hypothesis space. Results show that the agent's posterior beliefs converge to a single most probable model when combined with the Occam's razor decision criterion.
That being said, I have the following major concerns:
1. It is not entirely clear whether the restricted model class constitutes a genuinely novel structure learning approach or a regularised parameter learning scheme. Broader notions of structure learning — such as hierarchical depth or latent dimensionality — are neither addressed nor discussed as limitations.
2. Empirical studies are conducted only for the likelihood mapping. Although learning the full model from scratch is discussed, it is not tested, leaving a significant gap between the theoretical claims and the empirical validation.
3. The Bayesian model reduction framework is introduced without adequate explanation or intuition, making it difficult to follow even for a technically experienced reader.
4. The construction of the restricted model class is dense and hard to follow; additional exposition or a worked example would substantially aid comprehension.

Minor Concerns

• Missing full stop at the end of: "First, it draws a clear distinction between the unknown variables entailed by a generative model; namely, latent states, parameters and structure, c.f., (Kemp and Tenenbaum, 2008)"
• Typographical error at the start of: "The So, does planning with epistemic intentions provide a sufficient account of reasoning?"

Other questions for the authors

Introduction
• How are the "a priori preferred" outcomes constructed?
• Can a time-, context-, and experience-dependent mapping from observations to expected free energy be learned?
• Please clarify the claim: "one cannot learn to be curious" — is this a theoretical limitation of the current framework or a broader claim?
Active Inference, Learning and Selection
• How are the updates in the factor graph (Figure 2) derived, and what do the equality signs symbolise?
• Is there a missing subscript m on the calligraphic B in Equation 9?
Three Ball Paradigm
• How is the model space M constructed from different priors?
Conclusion / Scope
• The authors position the work in relation to the ARC-AGI-3 challenge, but given the restrictive assumptions made — a known generative model structure, a predefined hypothesis space, and discrete state spaces — it is unclear whether and

how the proposed approach could be applied to actual ARC-AGI-3 tasks. Could the authors elaborate on this?

(Remarks on code availability)

Version 1:

Reviewer comments:

Reviewer #1

(Remarks to the Author)
The authors have addressed my suggestions in a convincing manner.

(Remarks on code availability)

Reviewer #2

(Remarks to the Author)
Review of Active Inference and Artificial Reasoning: round 2

First, I want to thank the authors for engaging with my review so thoroughly and courteously. They have largely addressed my comments, and I would be happy to recommend the article for publication.
The main two points that the authors might find worth looking at, however, is commenting on the apparent small difference between when using the full expected information gain and that only for parameters and states but not model space; and commenting briefly on to which degree the simulation results are specific for the task or can be generalised (they probably relate to each other). Below, I respond to a few points; anything that I do not touch upon can be considered as fully addressed by the authors.

Clarity: structure of introduction
- I am happy for the authors to choose the structure they find the most useful here, and will leave it to the editorial team to judge if there is any reason to revisit this.

Limitations: only compatible with Bayesian model reduction
- Indeed, as the authors point out, the computational efficiency of their method is an advantage. I suppose I was thinking that the principle of including information gain in model space in the expected free energy should also be possible in the general case, where candidate models are not necessarily a subset of a full model and where Bayesian model reduction is not available. This would, it seemed to me, also be potentially useful, and therefore worth clarifying how well the approach here could be generalised to this case (or if it is strictly usable for the model reduction case). The authors should feel entirely free to decide whether they feel this is worth mentioning, however – I do not have any strong opinion here.
- I suppose I meant specifically that the efficiency of the model reduction approach comes at the cost of lower flexibility (re. the point above); and that the prioritisation of speed above flexibility might be motivated. Again, it would be completely acceptable if the authors would rather omit this point!

Limitations: the three-ball paradigm
- My apologies for being unclear here! The intention was just to ask to which degree the specific task (and accompanying generative model) matters for how much the added information gain in the expected free energy matters. In other words, the advantage of this depends on how much actions can actually change the informativity of observations (about model space). It seemed to me that this specific task might be one where the advantage is somewhat mild; I expect there would be other tasks where the difference would be more stark (and also where it wouldn't be there at all). This might be good to mention, just to ensure that conclusions here are not generalised too naively to other kinds of tasks.
The second part here was intended to highlight that the procedure of having flat preference priors during the model selection process is also a possibility due to the specific task; other tasks might require goal-directed behaviour early on, disallowing the behaviour that emerges here where the agent guesses rather broadly (to get as much information as possible) in the beginning.
In sum, perhaps the call was for briefly discussing or clarifying to which degree the numerical results here are specific to this version of the three-ball paradigm, and to which degree they can be expected to generalise.

Simulation methodology:
- I apologise – indeed this comparison is in what is now Figure 7. It is good that it is included in Figure 8 now, as well (I expect this figure was what motivated this comment from me).
- A brief note to Figure 8: the new version does not have any titles for the rows, so it is only by reading the figure text that it becomes clear what the different rows represent. Perhaps such titles could be added – if the authors find it useful.
- Finally, it seems that the difference between when including parameter and including model space information gain in addition is quite small (if I read Figure 8 correctly; about half the games have worse performance, but the other half have

better). It would probably be useful to comment on this; I suspect this would look different in some other tasks, where strategies that lead to higher expected parameter information gain do not necessarily lead to higher model space information gain (see the previous comment). The authors might sketch, for future work, tasks where this would be the case.
- Also, the figures are indeed now much prettier!

Sequence of cues:
- I clearly misunderstood how “sequence of cues” was intended to be read. My apologies! The readers can consider whether they think my confusion is indicative that other readers might also be confused or not, and accordingly judge whether they want to clarify further.

Language and minor points:
p. 5: “…, well chosen, …”: the commas here seem awkward, although perhaps the problem is my ignorance
Table 1 (glossary): “different kinds of variable”: should be plural

CODE:
- The code runs and produces the figures from the article.
- There is no README or similar to run the code; users must install MATLAB, and the development version of SPM that the script is embedded in, themselves.
- The code is clearly structured and with good, high-level commenting
- Variable names are highly abbreviated, which can make reading the code a bit difficult to read.
- There is a NOTES section in the end of the script, after the return statement. It is unclear whether one should use this. It seems to include code for generating figures, but also for a type of Jensen’s inequality implementation.
- In several places, the code makes reference to specific paths to personal folders. These are not necessary for running the main part of the code, but might conflict if one tried to run the NOTES part.
- Notably, the code relies on a specific in-development version of SPM (the code cannot be run with older versions of SPM). This means that, as SPM changes, so might (in principle) the outcome of the code. For full reproducibility, the authors might consider either having a timestamped/immutable repository with the code and all the necessary dependencies, or at least pointing specifically (in the article) to the specific version (i.e., the specific commit) of the SPM development repository that is used for the article. There might be other good practices for simulation reproducibility that I am not aware of, which the authors might be prefer; I do not have strong opinions on this.

(Remarks on code availability)
See the main response to reviewers for details.

Reviewer #3

(Remarks to the Author)

I thank the authors for their thorough and thoughtful revisions. The additional discussion on broader structure learning challenges, the new Figure 4, the relocated explanation of Bayesian model reduction, and the various clarifications throughout the manuscript have all meaningfully improved the paper.
I am largely satisfied with the responses to my concerns. My one remaining reservation concerns the distance between the theoretical ambition of the paper and its empirical scope. I appreciate the authors' candour that "active reasoning, as described in this paper, only addresses a small part of this challenge," and I recognise that likelihood mapping discovery is the natural entry point for rule learning under active inference. However, I would encourage the authors to consider whether the introduction of the manuscript adequately signal this narrower scope to readers who may otherwise expect a more general structure learning contribution. An adjustment here — perhaps foregrounding the focus on rule discovery more explicitly — would help calibrate expectations and let the genuine contributions of the work stand on their own merits.

(Remarks on code availability)

Open Access This Peer Review File is licensed under a Creative Commons Attribution 4.0 International License, which permits use, sharing, adaptation, distribution and reproduction in any medium or format, as long as you give appropriate credit to the original author(s) and the source, provide a link to the Creative Commons license, and indicate if changes were made.
In cases where reviewers are anonymous, credit should be given to 'Anonymous Referee' and the source.
The images or other third party material in this Peer Review File are included in the article's Creative Commons license, unless indicated otherwise in a credit line to the material. If material is not included in the article's Creative Commons license and your intended use is not permitted by statutory regulation or exceeds the permitted use, you will need to obtain permission directly from the copyright holder.
To view a copy of this license, visit https://creativecommons.org/licenses/by/4.0/

Reviewer #1:

This paper is a mathematically interesting contribution that extends expected free energy with an explicit information gain over models, alongside the usual gains over states and parameters. That is a genuine strength: the authors do not merely argue that reasoning can be cast as model selection, but provide a concrete variational framework that integrates model comparison into active inference. A second strength is the clean organization across timescales, separating inference, learning, and model selection in a way that is conceptually elegant and internally consistent.

The practical side is also handled well within the chosen setting. The three-ball task is simple, but it is genuinely partially observed, so the agent must decide what to inspect before it can answer correctly. This makes the example more meaningful than a standard fully observed toy problem. The ablation results are another clear strength: they show that information gain over models is not just a theoretical addition, but materially improves discovery speed and overall task performance.

Many thanks for this excellent summary.

My main mathematical concern is that the method still depends heavily on a hand-crafted hypothesis space. The variational derivations are elegant, but much of the real difficulty is pushed into specifying the right set of candidate models in advance. In that sense, the framework is strongest once the correct model class is already available. I also find some design choices insufficiently principled, such as the thresholding and count-scaling heuristics, which seem more pragmatic than theoretically grounded. Would it be possible to assume less prior knowledge about the models? What prior knowledge would be plausible from a neuroscience perspective? I encourage the authors to consider those two questions.

Thank you for this. Yes, you are correct to highlight that one of the core problems here is how to determine a principled set of candidate model structures, which then allow for efficient variational inference procedures. In this sense, one of the core contributions of this paper is analysis of the kinds of symmetries—implicitly, priors over model structures—that allow one to nuance the problem of structure learning.

On the thresholding using Occam's razor, this is not strictly necessary or in any way fundamental to the theory developed here. We have added the following to make this clear:

> "While this thresholding can be easily motivated from a pragmatic perspective (there is no point continuing to evaluate alternative models when there is a clear winner), it is not fundamental to the underlying theory, and one could substitute this with alternative thresholds or omit the thresholding altogether. In practice, a Bayesian model average under a set of models in which only one is plausible would achieve a very similar outcome."

On the count scaling, we assume you are referring to the comments below Equation 13 about practical ways of suppressing or enhancing novelty seeking behaviour? We hope it is clear as written that this is a choice one can make in practice. To explain the principled underpinnings of this, we have added (in bold):

> "In practice, one can multiply the Dirichlet counts in the selected model by some arbitrarily high number (e.g., 512), to suppress novelty seeking. **This works because this high scaling implies high prior confidence and therefore little parameter uncertainty to resolve.**"

Regarding your first question: yes, one could omit some of or all the assumed priors that help keep the model space relatively small. Of course, doing so commits to a prior that all possible rules are equally

plausible—itself an implausible assumption. The less prior information given, the less tractable the problem. The challenge here is in identifying a set of plausible priors that might be generally applicable. Arguably, many of the advances in artificial intelligence over the last decade depend upon the imposition of the right set of priors (e.g., the second order non-Markovian structural prior imposed by attention heads in large language models) to draw more powerful inferences from fewer datapoints. In short, we see the method for determining plausible priors as a positive theoretical contribution of this paper that helps ensure the problem is tractable, and have emphasised this in a more explicit list of contributions at the start of the paper:

> "The specific theoretical contributions of this paper are as follows:
>
> - One can engage in active structure learning by using expected information gain about model structures to guide epistemically motivated behaviour. This dramatically extends the applicability of active inference to the domain of structure learning and offers an efficient means of data-selection.
> - Use of Bayesian Model Reduction techniques allows one to do this efficiently by evaluating a model average and then *post-hoc* selecting between the models contributing to that average without having to reinvert the original model.
> - One can further improve the efficiency of this procedure through a principled formulation of the model space to be evaluated. This will vary somewhat between different applications. However, in general, all possible models are not equally plausible. We illustrate how one can exploit the symmetries implicit in sensor and action modalities to constrain this prior model space and thereby ensure tractability."

Having said this, we have taken care to emphasise that the space of models is not universal, and there is scope to either relax or tighten the space of priors:

> "Clearly, committing to the above (isomorphic) rules does not offer a universal way of specifying a model space. For example, we could consider the context state to be specified by another context state leading to 3rd order hypotheses. Or, we could have added a further constraint that each context state specifies a unique criterion state, leading to $3\times3! = 18$ possible rules. However, this would preclude simple (first-order) rules; i.e., a fixed criterion state. […] One could also consider constraints afforded by multiple choice factors to increase the size of the model space, if deemed plausible a priori. For example, we could have considered rules of the form in which a correct response can only be obtained when looking at the start location"

Regarding your second question we have extended the discussion to include implications for the neuroscience of reasoning. The part of this directly relevant to your question is as follows:

> "At a more fundamental level, there are some constraints built into the biological machinery we have for sensing and acting upon the world. These constraints support the use of symmetries in constructing plausible, and tractable, priors for the space of models. For instance, colour perception depends upon the same retinal cone cells whether they register a cue or a response. Tactile sensing of an object in a spatial location also facilitates motoric manipulation targeted at the same spatial location. More generally, our musculoskeletal effectors are paired with proprioceptive sense organs. This implies a degree of isomorphism between the responses one might make and the sensory modalities that report upon these, supporting models in which these isomorphisms are respected. Some symmetries are relatively generic [e.g., see (Friston, Da Costa et al. 2023) for an analysis of the need for self-organising systems to exist in minimally ambiguous environments, supporting sparse models] while others may need to be relaxed depending upon context. Ultimately, for a creature to be a successful rule-learner, they must hold a set of priors consistent with rule generation. The challenge for reasoning neuroscience is to determine the form these priors take."

My main practical concern is scalability. The paper demonstrates the approach on a carefully structured toy problem where the representation is largely known and only one key mapping must be inferred. That is a useful proof of concept, but it falls short of showing practical reasoning in more realistic settings with richer observations and much larger hypothesis spaces. Overall, I find the paper strong in mathematical formulation and clear in its practical motivation, but still limited by its reliance on manual model design and its narrow empirical scope. If the hypothesis space is much larger, could one rely on naive mean-field approximations in variational inference to keep the computations tractable? I recommend the authors to address the scalability issue.

We fully agree that scalability is vital here. Our suggestion here is that a solution to the problem of scalability—at least, in the setting of reasoning—is the use of generic priors to constrain the space of possible solutions. In short, as you rightly point out, the problem for scalability is the application of methods of model comparison to very large hypothesis spaces. One can solve this scalability problem if one can find ways to automatically constrict that hypothesis space to a more tractable size. Your suggestion on the use of mean-field methods is interesting here. We have added the following to unpack this along with the broader points on scalability:

> "Although our simulations concern a relatively simple problem, it is one that has been chosen because of its large hypothesis space (even under sparsity constraints, of size 2^{432}). This addresses a key problem in designing practical tools: scaling to situations in which there are a very large number of possible solutions to adjudicate between. The solution we propose here is to note that many (most) possible solutions are not plausible solutions. By imposing a series of priors that gradually restrict this solution space until the problem is tractable, one can address the scalability problem. Does this guarantee a correct solution? Clearly not. It may be that the priors chosen are overly restrictive and exclude an a priori unlikely, but ultimately correct, rule. As shown in Figure 4 **[new in this revision]**, the chance of inappropriately excluding a model increases with each successive symmetry that is imposed. In some situations, it may be appropriate to impose some, but not all, of these prior constraints—in other situations, it may be necessary to impose more. Each carries with it the balance between tractable inference in scalable settings compared to the possibility of excluding a 'true' model.
>
> Are there other approaches one could adopt in place of, or in parallel with, this approach to ensure greater scaling potential? One option might be to apply the same principles of factorisation we use for hidden states to the model space itself. If there are some clear dimensions along which families of rules can be defined, could apply standard variational methods (Beal 2003, Winn and Bishop 2005, Dauwels 2007) and assume a mean-field factorisation across these dimensions. In principle, this lets one iterate between model comparisons for each model-space dimension, rather than having to evaluate all possible combinations in a single comparison. The challenge in applying a method of this sort is in characterising the right dimensions along which to factorise a model, in which comparisons for each factor can be made approximately independently. However, the compatibility of approaches like this with the methods outlined in this paper offer an opportunity to mix methods and potentially achieve even greater gains in scalability."

Regarding automatic versus manual model design, we have added a reference to the following papers detailing ways in which one can automatically generate models of this structure to emphasise that, at least in principle, one does not have to start from a manually defined model:

> "…applications of active inference approaches that learn the sorts of models we have used here (Smith, Schwartenbeck et al. 2020, Friston, Heins et al. 2024, Friston, Da Costa et al. 2024). The challenges one has to solve when building a model of this sort include determination of the

appropriate hierarchical depth, the dimensionality of each state factor, the number of state factors, the number of plausible paths, and so on. These are challenging problems. However, in principle they can be addressed through a process of making a series of changes to a model (either to grow or reduce it) and by assessing the model evidence before and after that change."

We hope the above revisions and responses are what you had in mind.

Reviewer #2:

In this article, the authors propose an efficient method for extending the expected information gain component of the expected free energy, used as objective function for selecting action ins active inference, so that is also includes the expected information gain about model structure. They show in simulation studies that this method, which applies specifically when using Bayesian model reduction under fully discrete partially observed Markov decision process generative models, leads to increased sample efficiency as compared to only using information gain about states and parameters. The paper is well-written and consistent, and the introduced methodology is certainly relevant for the use of active inference models in the various contexts where they are used, including in artificial intelligence and for computational phenotyping such as in computational psychiatry. I am therefore happy to recommend this work for publication. I do, however, have a set of minor recommendations that I hope can contribute to improving the manuscript, such as regarding clarity, discussion of limitations, situating the work in the broader literature, and regarding the simulation methodology. Unless otherwise is mentioned, these should be seen as optional.

Many thanks for this evaluation and for your detailed and thoughtful appraisal of the manuscript.

Clarity: technical extension or model of reasoning.

Throughout the article, it can feel, to me, somewhat unclear whether the aim of the work is to technically extend the active inference framework, or to provide a computational implementation of a theory of (human) reasoning. This should be made clear from the beginning.

If the aim is indeed to provide a model of reasoning, the paper should also include at least a brief overview of the literature already engaging with this goal, beyond the (often somewhat casual) use of the term in language models. It should also be justified why, exactly, being sensitive to information gain in model space when choosing where to look constitutes a model of reasoning. Finally, it would be useful to have at least a brief sketch in the discussion of how one might test this account of reasoning empirically in the future, such as by collecting behavioural data from reasoning tasks and investigating whether the augmented information gain term improves the ability of the model to capture the behaviour.

If the purpose is rather to technically extend the active inference framework to be sensitive to information gain in model space, and the term "reasoning" is used more casually here, the authors might make this more explicit early in the article.

Thanks for this. We have added the following to the introduction to make clear that this paper concerns *artificial* reasoning, acknowledging that there is a rich literature on human reasoning that one could consider applying this work to:

"Our focus in this paper is on *artificial* reasoning, as opposed to an explicit neurobiological theory of reasoning. Having said this, the theoretical contributions of this paper rest upon a biologically grounded framework derived from neuroscience (Shipp 2016, Friston, FitzGerald et al. 2017, Parr and Friston 2018), and it will be interesting to see how this applies more generally in cognitive neuroscience. Although we defer a more thorough treatment of this interesting and richly studied topic [see (Krawczyk 2018) for an overview] to future work, we will briefly touch upon this in the discussion. We also note that a core contribution of this paper is the use of action to sample a small number of, well chosen, datapoints sequentially to best resolve uncertainty about model structure. This is much like the foveal sampling that natural selection has identified as an efficient means of solving the same problem. *Reasoning*, as we use the term here, is the process of optimising a model structure to effectively discover rules. The role of active inference is to combine beliefs about model spaces—with priors capturing the relevant symmetries and constraints—with information seeking through action."

We have also added a paragraph to the discussion as you have suggested:

"Finally, although our focus has been on artificial reasoning, we conclude with some brief observations on human reasoning (Krawczyk 2018). There are a broad range of definitions, and categories, of reasoning in cognitive science. However, common tasks used in human behavioural experiments are based upon inference of rules, such as the Raven's matrix task (Raven 1941). Here, as in our setting, the challenge is to take a series of cues and to infer from these a rule that allows one to determine the likely next cue. This endorses our focus upon rule learning as a core element of reasoning. Anatomically, there is evidence that reasoning of this sort relies upon a lateralised, primarily left-hemispheric, frontoparietal network with basal ganglia involvement (Prado, Chadha et al. 2011). The basal ganglia element of this is particularly relevant here, in that these subcortical nuclei are often closely linked to action, with lesions associated to neurological disorders of action initiation (Albin, Young et al. 1989, Nambu 2004, Marreiros, Cagnan et al. 2013, Shine, Matar et al. 2013, Cagnan, Mallet et al. 2019). One could read this evidence as an endorsement of *active* reasoning, that the same structures involved in generating behaviour—motivated by preference satisfaction and information seeking—also support rule learning. One could assess whether our account of artificial reasoning might also serve as a (partial) theory of human reasoning through asking human participants to perform the 3-ball task, and by determining which of the patterns shown in Figure 7 **[previously Figure 6]** best matches their behavioural trajectory.

At a more fundamental level, there are some constraints built into the biological machinery we have for sensing and acting upon the world. These constraints support the use of symmetries in constructing plausible, and tractable, priors for the space of models. For instance, colour perception depends upon the same retinal cone cells whether they register a cue or a response. Tactile sensing of an object in a spatial location also facilitates motoric manipulation targeted at the same spatial location. More generally, our musculoskeletal effectors are paired with proprioceptive sense organs. This implies a degree of isomorphism between the responses one might make and the sensory modalities that report upon these, supporting models in which these isomorphisms are respected. Some symmetries are relatively generic [e.g., see (Friston, Da Costa et al. 2023) for an analysis of the need for self-organising systems to exist in minimally ambiguous environments, supporting sparse models] while others may need to be relaxed depending upon context. Ultimately, for a creature to be a successful rule-learner, they must hold a set of priors consistent with rule generation. The challenge for reasoning neuroscience is to determine the form these priors take."

Clarity: technical terminology

The article (and the active inference framework) uses a quite extensive technical terminology that is quite specific to its field, but also sometimes varies between articles and authors in it. It would be quite helpful to have an overview of this early on, perhaps in a small box. Here I mean terms like "state", "factor", "observation", "modality", "path", "policy", "action", "parameter", "belief" and so on. This is especially relevant because different terms are used to describe the dimensionalities of states, observations and actions (i.e., factor, modality, and I am unsure what the value of a single timepoint for a single dimension of an action is called). This could also include the mathematical annotation for the different terms. Establishing such an overview might be nice for the active inference field in general.

Thanks for this suggestion. We have added the following glossary table to help with this:

Term	Description
State	Latent or hidden states are unobserved variables from which observations may be predicted. These are typically dynamic and may evolve from one time-step to the next as governed by transition probability matrices. For the purposes of this paper, they are assumed to be categorical variables, each taking on one of a countable number of discrete values.
Observation	Observations are the sensory data (or outcomes) one can use to draw inferences about the states, models, and parameters that cause them. Again, for the purposes of this paper, these are assumed to be categorical variables.
Factor	States may be structured according to distinct factors. This greatly simplifies a state-space in that one can represent different kinds of variable independently of one another, as opposed to having states representing all possible combinations of variables. For instance, one might represent one's location along discretised North-South and East-West factors, rather than having a single state factor that can take on the role of any combination of these.
Modality	We will use the term 'modality' as the observational analogue to state factors. This reflects the notion of sensory modalities (vision, audition, etc) in biology.
Path	Paths index, for each state factor, sets of transition probabilities (matrices as subtensors of transition probability tensors) that determine alternative probabilistic trajectories for that variable.
Policy	Policies are combinations of controllable paths and therefore can have implications for the paths followed across multiple state factors.
Action	An action is the effect of selecting among alternative (controllable) paths at a given point in time.
Parameter	Parameters are a subset of latent states that do not vary dynamically (although inferences about parameters will do so). Typically, in this setting, parameters are Dirichlet distributed variables that determine prior or conditional probabilities of states, paths, and observations.
Belief	Beliefs are probability distributions describing states, parameters, and model structures. When used without qualification, they will tend to refer to (marginal) posterior probabilities.
Inference	Inference is the process of optimising (i.e., updating) beliefs about states to extremise variational free energy—thereby achieving (inference to) the best explanation as to the causes of one's observations.
Learning	Learning is inference about either parameters (parameter learning) or models (structure learning).

The authors use "path" to refer to a single dimension of the action over time (distinguishing it from a "policy", which refers to all the different action dimensions). In other fields, "paths" would not necessarily be considered one-dimensional, nor would they only refer to actions. Perhaps there is a better term than "path"? In any case, it is not clearly introduced before it is used in the figure text for Figure 1, which I expect would be confusing to many readers.

Sorry not to have been clear about this. We did not mean to imply that paths are one-dimensional at all. We hope the description in the table above helps to clarify this.

The authors early on use the term "learning" to refer specifically to parameter learning, and distinguish it from "selection", which refers to model selection. However, they also call the model selection "rule learning", and mention "learning a generative model". To be consistent in their terminology, they might change "learning" to "parameter learning", and use "learning" for parameter and model learning in general; or they might not use the term "learning" for the Bayesian model selection, despite the fact that this is often called "structure learning" in other fields. Finally, if the authors here intend for "structure learning", "rule learning" and "learning a model" to be synonymous, they might make this explicit; if not, they might clarify the relations between these terms.

Yes, we fully agree. To keep the link with structure learning, we adopt your first suggestion and make clear where we are referring to parameter learning versus structure learning specifically.

The authors call the kind of Bayesian model selection used in the paper a post-hoc approach. They might early on make clearly exactly what is meant with this. What makes it post-hoc?

We have added the following to address this:

> "By *post-hoc*, we mean that this is a form of model selection that takes place after data assimilation into an existing model. This is because Bayesian Model Reduction allows for assessment of the evidence for—and posteriors one would have obtained—under a 'reduced' version of the model, without having to repeat the process of model fitting. However, it requires the posterior parameters estimated from a 'full' model to be able to do so and can only be applied *post-hoc.*"

Clarity: formal details

It might be useful if the digamma function and its role in the formalism is introduced briefly, to users who are not familiar with it.

Of course. We now say:

> "The digamma function—the logarithmic derivative of the gamma function—appears here as it affords a concise representation of the expectation of (log) parameters under a Dirichlet distribution."

If I understand correctly, a model-average of the various possible models is used in the beginning, until a single model is committed to. During this pre-commitment phase, parameter learning is done across all the possible models, which is feasible because it still only has to be done once (under this model reduction scheme) – the parameters can be reused, so to speak, because the different models are versions of the full model and the updated parameters are the same across models. Is that correct? Or does parameter learning not begin until the model has been selected?

Since I was a bit in doubt, perhaps it is useful to clarify this in the article. Finally, it might be discussed whether there are formal limitations to this approach to Bayesian model reduction, at least if my first interpretation is correct. Is it an approximation, or would it give the same results as if one used the (computationally heavy) approach of calculating the variational free energy for each sub-model separately and compared their model evidences?

Yes—your understanding here is essentially correct. To make this explicit, we have added the following summary just before introducing the 3-ball paradigm:

> "One can understand the procedure outlined above as follows. One begins by learning the parameters of a single model constructed via a Bayesian model average of all plausible ('reduced') models. Bayesian model reduction proceeds by comparing the learned parameters with the reduced priors associated with each of the reduced models to efficiently compute their evidence, until a single model gains overwhelmingly more evidence than the others, enabling commitment to this model."

Regarding Bayesian model reduction, we were reluctant to devote a lot of space to discussing this technique, which has been extensively unpacked elsewhere and is already in widespread use in model-based analysis of timeseries (functional neuroimaging) data. However, we have added the following to briefly answer your questions:

> "Bayesian model reduction gives the exact difference in model evidence—and reduced model parameters under specific conditions—when a reduced model differs from the full model in its priors. This holds when the posteriors under the full model are exact, and the priors are conjugate to the likelihood of the full and reduced models. When these things are approximately true, the estimates are themselves approximate. For readers interested in the details and applications of this technique, please see (Friston, Litvak et al. 2016, Friston, Parr et al. 2018)."

Clarity: structure of introduction

The section titled "Active Inference, Learning and Selection" has some overlap with the introduction, and some of it would make reading the introduction easier. Section such as p. 3 starting with "Technically, variational free energy minus" would perhaps fit better in the "Active Inference, learning and selection" section, for example. The authors might consider if there is a way to streamline this first part of the article more.

Thanks for this suggestion. While we agree that there is some overlap, we felt on balance that it would be useful to have the broader and less technically detailed information left in the introductory section. We did consider moving the paragraph you have indicated into the later section but found that this disrupted the logical sequence set out in the introduction and meant some concepts were introduced without suitable definition and motivation in subsequent paragraphs. However, we are open to revisiting this if you and the editorial team feel this would be helpful.

Clarity: overview of contributions

The paper seems to make several, distinct, and increasingly specific, contributions. One is treating the choice of model structure as a random variable in its own right, and including the expected information gain for this variable in the expected free energy. Another is to apply this specifically for the already introduced case of Bayesian model reduction used here, where submodels of the full model can be specified by setting parameter values to 0. A third is to provide a manageable subset of models for the three-ball task (by imposing various sparsity constraints) which allows using the above method in practice. A final contribution is to provide an Ockam's Razor approach where the agent moves from having a belief distribution about which model is true to committing to using only one model, only after which it has preferences about its observations. Giving a clear overview in this fashion early in the article might be useful for readers to understand where the contributions are, and how they depend on each other.

This is a very helpful suggestion. We have added the following to the end of the introduction:

> "The specific theoretical contributions of this paper are as follows:
>
> - One can engage in active structure learning by using expected information gain about model structures to guide epistemically motivated behaviour. This dramatically extends the applicability of active inference to the domain of structure learning and offers an efficient means of data-selection.
> - Use of Bayesian Model Reduction techniques allows one to do this efficiently by evaluating a model average and then *post-hoc* selecting between the models contributing to that average without having to reinvert the original model.
> - One can further improve the efficiency of this procedure through a principled formulation of the model space to be evaluated. This will vary somewhat between different applications. However, in general, all possible models are not equally plausible. We illustrate how one can exploit the symmetries implicit in sensor and action modalities to constrain this prior model space and thereby ensure tractability.
>
> Each of these contributions is supported by both theoretical and numerical analyses."

Limitations: specific generative model type

The authors use a somewhat specific type of generative model here, namely a discrete state-, observation-, action-, time- and model-, but continuous parameter-, space partially observable Markov decision process. Only laten states have dynamics and depend on their own previous state; observations only depend on states, and parameters have no dynamics at all. In addition, it is only transition models parameterised by B that depend on actions (and not the observation model parameterised by A, nor the model space). This model family is of course ubiquitous in the active inference as generative model, but it might still be useful to clarify briefly the limiting assumptions it makes in general. More specifically, it would be useful to discuss whether the methods introduced in this article also apply to other types of generative model, such as continuous state space models.

In the discussion, we have added:

> "While this sort of model is relatively generic, dealing with categorical (and discretised continuous) data, one limitation of this style of model is that it can be very inefficient to represent high-dimensional continuous systems in this manner. This is because the degree of discretisation required can mean that one needs categorical states with very large numbers of possible values. This raises the question as to whether the methods outlined here can be applied directly to continuous state-space models that afford more efficient representations of such systems. In principle, the answer is yes, in that Bayesian Model Reduction—under a Laplace approximation—has been applied extensively to continuous systems (Friston and Penny 2011) and one can simply formulate information gains for such systems (Koudahl, Kouw et al. 2021, Parr, Friston et al. 2024), at least, to a reasonable approximation. However, the outstanding challenge to make this practical—for continuous models—is in identifying a set of candidate paths, as opposed to a continuum of possible paths, along which expected information gain should be evaluated. While plausible, this is less straightforward than in the categorical domain as presented here."

It might also be useful to clarify to which degree further limiting assumptions are imposed by this type of Bayesian model reduction and calculation of the model space expected information gain. In its current form, it seems to be incompatible with temporally deep planning. Is it also incompatible with

hierarchically deep generative models? It does seem compatible with counterfactually deep generative models. Is it compatible with sophisticated inference or inductive inference? Would it interact in any relevant way with either of the above?

In principle, we do not see any reason that this approach would be limited by or incompatible with the methods you have outlined—except where specifically mentioned already in the text. We would be reluctant to speculate here and expect that numerical studies of these potential interactions would overburden this paper.

Limitations: only compatible with Bayesian model reduction

The authors might point out that the method they use is here is compatible only for use with the type of Bayesian model reduction they use (although not the general principle or including information gain about a random variable controlling the choice of model). This of course has advantages, as the authors mention, such as making the calculation highly efficient.

We were not sure that this is a limitation. Isn't this a positive contribution, giving a method by which an otherwise computationally intensive procedure can be performed efficiently? We hope that it is clear that the method we outline is dependent upon Bayesian model reduction.

First, the authors might motivate this requirement for speed a little; is it computationally infeasible to use other methods, or is the speed primarily relevant for applications that are highly speed-sensitive?

We have rarely been asked to justify why it is beneficial for computation to be efficient! To answer this, we have added:

> "Fast and efficient inference is important both in biological and artificial systems in conserving energy and ensuring timely action in a dynamic world. This is increasingly recognised as important for sustainability in the space of artificial intelligence (Henderson, Hu et al. 2020, Patterson, Gonzalez et al. 2021, Rillig, Ågerstrand et al. 2023)."

In addition, the authors might consider to which degree this Bayesian model reduction approach is plausible as a model of (human) cognition. Is it likely that humans would separately evaluate a model evidence for a pre-specified set of submodels? 81 models still seem many, at least for it to be done consciously. Additionally, this method require the model space to be fully pre-specified. In which cases could this be expected?

We fully agree that it is unlikely that humans consciously evaluate and select between 81 models and hope we have not left readers with this impression. In fact, the use of Bayesian model reduction based upon a model average means that one never needs to explicitly evaluate all these models through full inversion. As demonstrated in the simulations, the sequence of events is much closer to a gradual pruning of models in parallel until reaching a point at which one model dominates the Bayesian model average.

Regarding modelling human cognition, please see our response to your question above and the new paragraph added to the discussion. There are interesting issues (not covered in this paper) that attend the kind of artificial reasoning considered here. In terms of neurobiological implementation, this would be the kind of process associated with synaptic homeostasis and sleep; namely, a dropout of redundant connections that would not be recognised at a declarative or propositional level (please see below).

Limitations: rule learning, not structure learning

In this article, the authors focus specifically on rule-learning, which is arguably a special case of the much broader task of structure learning. In this context, the authors use a series of constraints to a priori reduce the space of possible models. This includes (if I understand correctly) assuming that a) a set of latent states only can result in a single feedback outcome, b) that rules are always a combination of an action and a single criterion latent state, and c) that a criterion laten state is fully determined by a single latent context state. These assumptions are of course appropriate for this version of the three-ball paradigm, but do not apply even to slightly different versions of the task (such as if the correct answer depended on more than a single position). The authors should make these limitations explicit early on (they are stated, but are somewhat difficult to read). A visual depiction of how these constraints limit the space of possible models would be quite useful, I think. The authors might discuss to which degree their approach might generalise to other cases, and discuss to which degree this furnishes a generally applicable way to a priori limit the model space. Finally, they might discuss whether a human participant is likely to approach this task with the same constraints or not.

Yes, rule-learning is an example of a structure learning problem. We have defined a rule as follows:

> "a unique mapping from every combination of latent states to some response; e.g., correct or incorrect. This could be a minimal definition of a rule."

We have also discussed the limitations you highlight explicitly as follows:

> "Clearly, committing to the above (isomorphic) rules does not offer a universal way of specifying a model space. For example, we could consider the context state to be specified by another context state leading to 3rd order hypotheses. Or, we could have added a further constraint that each context state specifies a unique criterion state, leading to $3\times3! = 18$ possible rules. However, this would preclude simple (first-order) rules; i.e., a fixed criterion state. From the perspective of the contextual rules, a first-order rule simply means the criterion state is specified when the context state is in the first level OR the second OR the third, and so on. […] One could also consider constraints afforded by multiple choice factors to increase the size of the model space, if deemed plausible a priori. For example, we could have considered rules of the form in which a correct response can only be obtained when looking at the start location."

Thank you for your suggestion of a visual depiction. We agree that this would be useful in helping make the textual description more intuitive and transparent. We hope the following is what you had in mind:

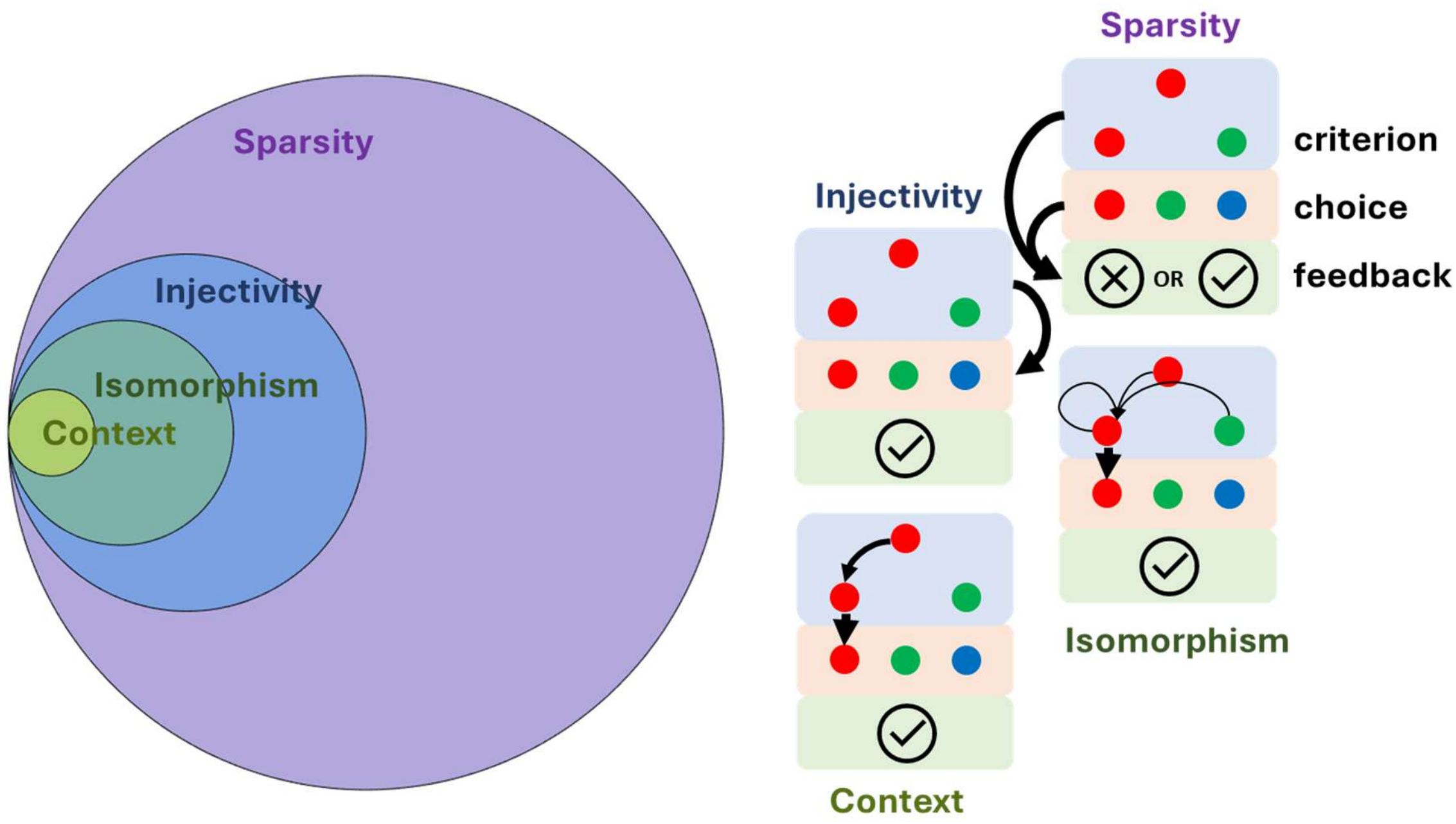

Figure 4: plausible rules. This figure is designed to give a graphical representation of the series of steps taken to limit the space of models used to those in which only plausible rules are in play. The graphic on the left shows the relative number of plausible rules (with the area of the circles scaling with the log number of rules) following application of each new constraint. The graphics on the right illustrate the kinds of rules included in each constraint. The initial sparsity constraint only allows for rules in which the feedback predicted is definitively positive or negative for any combination of choice and criterion states. The injectivity constraint says that, when the feedback is positive, there is an injective mapping from criterion to choice states. The isomorphism constraint says that one of the criterion factors that contains the same annotations (i.e., 'red', 'green', 'blue') as the choices. However, the specific factor (left, centre, or right cue) is here determined by some unknown context which may depend upon all three. Finally, the context constraint says that one of the criterion states (shown here as the centre state) provides all the contextual information required to determine the relevant criterion factor (shown here as the left state) that injectively and isomorphically maps to the choice state that ensures the feedback is positive. Please see the main text for more detail and pseudocode illustrating this.

Limitations: the three-ball paradigm

The authors might discuss the limitations imposed by using the three-ball paradigm in their simulations. It is for example perhaps a task where the information in model space is quite strongly dependent on the set of cues given to the agent, and arguably less dependent on the action the agent takes – in other words, I wonder if it is a task where the effect of including model space expected information gain is smaller than it might be in a task where action very strongly determine the effect of information one gets about model outcomes. The three-ball paradigm is also a task where, it seems, a main avenue for model space information is to make guesses given the cues a lot (as is seen in the simulation), which quite starkly impacts the pragmatic gain under non-flat preference priors (probably why flattening the preference priors is necessary in the beginning). One could easily imagine other tasks where it is possible to have preferences also during the model selection process.

We were not sure we fully understood what is being asked for here. The results of our simulations appear to show that solving this task is usefully benefited by model space expected information gain. However, we are sure you are right that there will be other paradigms for which this could be useful.

Limitations: Occam's Razor to one model

The authors use what they call an Ockam's Razor approach, where the agent has an initial phase where it does Bayesian model comparison, until the Bayes factor reaches a threshold that triggers committing fully to a single model. Only after this does the agent have preferences for the observations it gets.

The authors should discuss this modelling in more detail, and discuss the alternatives. First, what are alternatives? Arguably, a simpler approach is to just to use a Bayesian model average of the different models when planning, for example. If the posterior in model space is sharp, it seems this should become identical to the single-model approach.

Thanks, we now say:

> "While this thresholding can be easily motivated from a pragmatic perspective (there is no point continuing to evaluate alternative models when there is a clear winner), it is not fundamental to the underlying theory, and one could substitute this with alternative thresholds or omit the thresholding altogether. In practice, a Bayesian model average under a set of models in which only one is plausible would achieve a very similar outcome."

Second, what are the justifications for the approach in the paper? Is it primarily the lessened computational load of committing to a single model? Is it informational, that averaging across models will lead to unidentifiability in state inference, for example? Or is it motivated by an account of (human) cognition, where people necessarily commit to a single hypothesis before acting on it? Or is it rooted in other principles from the Bayesian mechanics that active inference rely on ?

It is the first of these. We hope our earlier edit in relation to your question about the benefit of speed helps to resolve this.

Third, what are the limitations of the method? It is for example not possible to change one's mind after committing to the model, even if strong evidence against it is provided afterwards. Fourth, the authors might discuss the fact that they use a threshold for choosing the model (as opposed to a continuous belief change, which is often presented as an advantage of Bayesian methods over other approaches). The choice of threshold seems somewhat arbitrary; would the authors expect to later let this threshold depend on a component of the generative model(s), such as a cost of being wrong in model space? Is there a way to derive this threshold in a principled manner? Would the threshold be context-sensitive?

There are a wide range of approaches one could take here, as you rightly point out. We would suggest not overinterpreting the thresholding and hope the additional text detailed above helps to explain why. You are correct to say that once one has committed to a model, it is impossible to then change one's mind. However, the purpose of the threshold is to ensure that one must reach overwhelming evidence in favour of one model, such that the chance of later having to change one's mind is very low.

Finally, the authors might discuss and justify the choice of letting the agent have flat prior preferences before committing to a model.

This is just for simplicity as the focus of this paper is on information seeking. Starting without this simplification, one ends up with a longer time before crossing Occam's threshold, as one would expect when there is a tension between information seeking and preference satisfaction. However, it is an

interesting question as to whether one could formulate a model in which this kind of threshold, and the accompanying move towards definitive preferences, might emerge naturally. We have added a footnote to the following to suggest one way this could be achieved:

> "At this point, informative preferences are adopted[7] and the agent plans, with greater confidence, two steps into the future. This point can be regarded as an 'aha moment' or the dénouement of some data gathering that licences a commitment to a particular hypothesis."

> "7. **The explicit separation between information seeking and preference satisfaction phases here is somewhat heuristic and may seem at odds with the spirit of active inference accounts of the natural move from exploration to exploitation that emerges from the balance between contributions to expected free energy (Friston, Rigoli et al. 2015). The reason for imposing a more explicit distinction here is that our focus is upon the information seeking elements, and this is more cleanly demonstrated when one suppresses preferences. Without this simplification, it takes longer to reach Occam's threshold. It is interesting to consider whether there may be a form of generative model in which this distinction emerges in a more principled manner. One answer to this would be to make use of a hierarchical (deep) model in which the higher level jointly predicts the precision of beliefs about the model space [e.g., (Hesp, Smith et al. 2021)] and the precision of preferences. One would expect that, as precision (i.e., confidence) about the model increases, evidence accumulates for a state of affairs in which preference precision is higher."**

Background: other kinds of structure learning

The Bayesian model reduction scheme used here is of course a special case of Bayesian model selection and structure learning in general. It could be useful if the authors situated their work somewhat within the larger field of Bayesian model selection, especially to discuss to which degree expected information gain could be calculated within other approaches. Would this approach for example be useful in the context of causal discovery, as a way to select informative actions about causal connections in a generative model (such as in Dynamical Causal Modelling)? Could it be used for model comparison where it is not the connections but different dynamics that are compared instead (such as is often done when modelling continuous state space phenomena). Is there a reason why priors in model space must be flat, beyond the use for the specific task here where we assume the agent has no previous knowledge about rules? Could it be combined with traditional methods that use (approximations of) the model evidence to select between models? Since a random variable controls the model, could this random variable have dynamics (i.e., a game where the rule changes, so that a different model becomes true at some point during the game)?

Again, we would be reluctant to devote much more space in this already long paper to rehearsing questions about Bayesian model reduction more generally or to speculate as to what would happen if it were applied in other domains. However, we note that it used widely in many of the situations you describe and in fact powers the Parametric Empirical Bayes schemes used in Dynamic Causal Modelling. We hope our earlier comments and reference to some key papers unpacking this in more detail help to resolve this.

An obvious extension, which may seem closer to (human) reasoning, and which could benefit greatly from the sample-efficient information seeking behaviour provided by this article, would be in open-ended structure learning scenarios where a model is "grown" (i.e., where new causes are added and

removed iteratively), such as in Dirichlet process models. The authors might briefly discuss whether and to which degree it would be possible to extend their method to include these use cases in the future.

Thank you for this. We have added the following to the discussion to address structure learning more broadly:

> "Other advances that might help fill in the gaps here include broader perspectives on structure learning, ranging from generic schemes like hierarchical Dirichlet process modelling (Teh, Jordan et al. 2006, van Erp, Nuijten et al. 2025) through proposed neural implementations (Gershman and Niv 2010, Tenenbaum, Kemp et al. 2011) and applications of active inference approaches that learn the sorts of models we have used here (Smith, Schwartenbeck et al. 2020, Friston, Heins et al. 2024, Friston, Da Costa et al. 2024). The challenges one has to solve—when building a model of this sort—include determination of the appropriate hierarchical depth, the dimensionality of each state factor, the number of state factors, the number of plausible paths, and so on. These are challenging problems. However, in principle they can be addressed by making a series of changes to a model (either to grow or reduce it) and by assessing the model evidence before and after each change. This, of course, raises further problems, which is the way in which one determines the sequence of changes to test and, if within one's control, the curriculum of observations on which to train a model. Solutions to these problems will be necessary to develop the models on which active reasoning rests."

Finally, there is also other work where Bayesian model selection is implemented by having a random variable control the model, such as in hierarchical mixture models. I know these best from fields using other approximations, such as sampling, to Bayesian inference, but they might still be worth mentioning in this context. Finally, the authors might discuss whether they would, in general, consider Bayesian model selection a special case of Bayesian inference over a latent variable that select between different model structures, or if this is a specific approach used in this article that should be considered distinct from other kinds of Bayesian model comparison.

Thanks—it is exactly that. We have added:

> "One can interpret Bayesian model comparison as the application of Bayesian inference to a random variable that effectively weights elements in a mixture model. Model selection is the special case in which the inference about the mixture coefficient approaches zero entropy (e.g., when one knows with confidence which cluster a datapoint is sampled from). It is this interpretation that allows one to directly incorporate model identity as a random variable into expected free energy."

For your interest, Bayesian model reduction has been used in the spirit of a mixture model to link categorical models (like that used here) with continuous state-space models in active inference (Friston, Parr et al. 2017).

Simulation methodology:

The authors run several simulation studies to investigate the effect of adding the model space expected information gain to the expected free energy. They do not clearly show this effect, however, since they show the difference when removing expected information in both model and parameter space simultaneously. Since this is arguably the main result in the article, it should be shown clearly. Secondly, they run the simulation only using the same initial conditions, and with the same set of cues, which makes it difficult to know how well their results generalise. I would also strongly recommend addressing this.

Apologies, but we were a little puzzled by this comment. The first and second columns of plots in Figure 6 (now Figure 7) directly perform the comparison you suggest between models with expected information gain for models being the only difference between the two. We have copied this figure here to show where this appears. Please do let us know if this is still unclear and we will seek to improve the presentation. Regarding initial conditions, we feel it is important to keep these consistent here for validity of comparison between schemes. This is essentially starting from a condition of uncertainty, which is the natural initial condition of interest here. In case this had been interpreted as every trial being identical, this is not the case. The sequence of initial conditions across trials for a given choice of information gains (applied to states, models, and parameters) is randomly generated, but this same sequence is used in the comparisons with other choices of information gains.

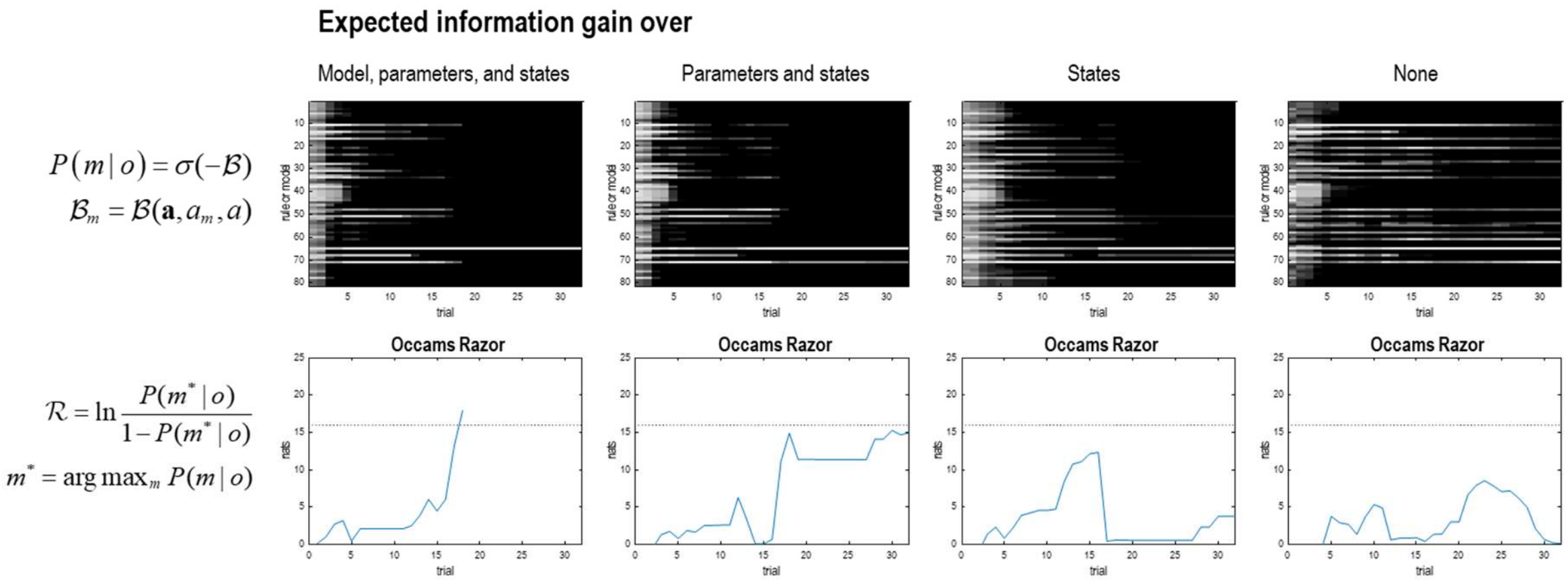

See the last section with the specific comments for more information.

Other suggestions for discussion

The authors might consider briefly sketching out how the current formulation would interact with postdiction (i.e., re-inferring past states when receiving new information). As I understand, it is already implicit in the message passing scheme used that beliefs about a given state depend on knowledge about later states. Information gain in model space would supposedly also help disambiguating beliefs about past states.

Thanks. We have added:

> "Interestingly, the foveation mechanism in our setup means that one needs a degree of 'postdiction' or smoothing such that inferences about past states can be recontextualised by new information. This would be particularly important in deep hierarchical models, where retrospective inferences about initial states determine ascending messages (Friston, Rosch et al. 2017) [although, see for contrast (Friston, Heins et al. 2025) in which access to the entire visual field at all times precludes a need for backwards smoothing]. The model selected through the procedure outlined here will have profound implications for this kind of postdiction—particularly as increased confidence in context state factors would retrospectively influence confidence in criterion states."

The authors might discuss briefly whether this augmented version of the expected free energy is also derivable from the free energy principle. I expect it to be, since it seems to be just treating the model as another random variable to have beliefs about.

You are absolutely correct here. We have incorporated this point into one of our edits detailed above and reproduced here for your convenience:

> "One can interpret Bayesian model comparison as the application of Bayesian inference to a random variable that effectively weights elements in a mixture model. Model selection is the special case in which the inference about the mixture coefficient approaches zero entropy (e.g., when one knows with confidence which cluster a datapoint is sampled from). It is this interpretation that allows one to directly incorporate model identity as a random variable into expected free energy."

The authors might discuss how this kind of model reduction might be implemented neuronally; would it be a sudden change in connection strengths between neurons, or some other mechanism? How does it relate to proposed neurobiological implementations of the Dirichlet parameters, which the model reduction seems to act on?

We have extended our previous comments on this to be more explicit:

> "It is usually implemented offline as a variational free energy minimising process of the sort that has been proposed to occur during introspection or sleep (Hinton, Dayan et al. 1995, Friston, Lin et al. 2017). **One could imagine this manifesting biologically through the dynamic formation and termination of connections between dendritic spines and axonal boutons (Deng and Dunaevsky 2005, Stettler, Yamahachi et al. 2006, Butz and van Ooyen 2013).**"

Language, formatting and minor suggestions

p.1 "exploratory, information-seeking actions": one could also add that "exploration" terms in many models are also essentially just added randomness or propensity to take actions not taken before, where it is here targeted specifically at sampling observations that are expected to be informative given current beliefs.

We hope this is implicit by including the phrase 'information seeking'?

p.1 "homologue of "thinking": the authors might justify this briefly, or at least indicate that it depends on what one means with "thinking". Indeed, thinking is sometimes defined as language-based or explicit/aware, neither of which are a priori compatible with the planning as inference account.

We have used this term in the context of 'system 1' and 'system 2' thinking which make no such commitments to purely linguistic forms of thought. We agree that this would be interesting to unpack in detail but suspect this paper is probably not the place to engage with this level of philosophical discussion (which we could not do justice to in the space available).

p.2 "one cannot learn to be curious": I would expect one could train a mapping from current beliefs and observations to the expected free energy – perhaps I am mistaken

This is a subtle but important point. We have added the following footnote to unpack what was implied here:

"[1]Although it is possible to amortise (i.e., learn), time-independent mappings from observations to posteriors over states, one cannot learn a time-dependent mapping from a posterior over states and parameters to expected free energy. This is because the mapping changes with learning. Heuristically, one cannot learn to do something one has never done before. Having said this, it is perfectly possible to learn epistemic habits (Friston, FitzGerald et al. 2016); e.g., sitting down to watch the evening news. However, one cannot learn to do something one has never done before."

p.2 "The So,": typo

Thanks—corrected.

p.3 "cover abstraction": the authors might clarify how exactly abstraction and reasoning are equated, and also why model space information gain sensitivity could be considered a type of abstraction.

We now say:

"…in the sense that a rule is an abstraction of the behaviour of a system, applicable to multiple specific instances of that system."

p.3 "under the partially observed": the authors might here and henceforth make clear that they refer specifically to discrete state, action and observation POMDPs, since the following formalism doesn't seem to apply to continuous formulations. They do mention this at times, but it might be nice to clear from the beginning.

We now say:

"…partially observed **(categorical)** Markov decision processes…"

Figure 1. "empirical prior": This use of the term seems to diverge from how it is used in Bayesian inference to describe priors chosen based on previous work and data. Since "empirical" seems to denote something dependent on empirical data, it seems somewhat odd to use it for priors that depend on other variables in the model. Perhaps there is a standard term for this in the broader Bayesian inference world? I suppose this is often what the term "hyperprior" targets, although this of course describe the random variables which the "empirical priors" depend on.
Figure 1. The optimisations in the bottom-right corner of the figure are not explained in much detail in the figure text, and only some of it in the main text quite a bit later (including the cursive l function of the E matrix, or the difference between the bold and non-bold matrix names). Perhaps giving a brief explanation here would be useful.

This a standard term that inherits from hierarchical Bayesian schemes, including Parametric Empirical Bayes. The reason for this is that inferences about the variables in the conditioning set of a prior (which one could call hyperparameters in the right setting, as you note), themselves drawn from empirical data, change the effective prior. As such, the prior is modified by empirical data.

Equation 1: Should there be a subscript T for the variational free energy F on the left side? I suppose this will be the F at a specific point in time, and the right hand side of the equations uses t subscripts.

We have added:

> "Clearly the free energy is a function of observations and a functional of the current beliefs held, and could be written as $F[o_\tau, Q(s_\tau, u_\tau, \mathbf{A}), P(\ldots)]$. However, to keep the notation concise, we omit these functional dependencies."

Equation 1: the dependence on the model m is explicated for the log model evidence p(o|m). I suppose the dependence on the model is also implicit in many of the other terms, however. The authors might consider making this consistent, and if not, stating clearly that omit the model dependency only in some terms. This is particularly relevant because this paper focuses specifically on model structure learning.

Yes, this is a good point. We have added:

> "We have conditioned the model evidence term explicitly upon a model, *m*, to emphasise its interpretation. However, all other terms are also implicitly conditioned upon the model."

Equation 1: when calculating the prior in the first line, the transition from s-1 to 1 is calculated relative to the action u at time t (not t-1). Perhaps I am mistaken, but I would expect that it is the action at the previous timestep that controls this transition, since the action at the current timestep hasn't happened yet.

Probably the best place to look if you are interested in the transition structure is Figure 1, which sets this out explicitly. The prior in Equation 1 is not the full transition probability and implicitly marginalises over the action at the previous point in time. However, we agree that having the current action in the conditioning set is misleading here and have now omitted this and included the previous action for clarity.

Equation 1: In the final line, in the term for calculating the complexity, I suppose the true posterior over states, actions and parameters will also be conditional on the previous action, and not just the previous state?

Yes, but as above this has implicitly been marginalised out.

Equation 2: The authors point out that they omit some parts of the full equation here. This is fine, but of course can make the mathematics more opaque. I would find it useful if they would include a fully spelled out version of this and other equations in the appendix.

Thanks—we have outlined in the text and provided references for the way this would be extended to accumulate along different paths for longer planning horizons:

> "Planning over longer time horizons simply involves accumulating the path integral of expected free energy over successive actions—or combinations of actions; i.e., policies—as described in (Friston, Da Costa et al. 2021)."

The other omission we assume you refer to here is the focus upon likelihood parameters only. We have added:

> "One could construct a more complete expected free energy through inclusion of other parameters (e.g., transitions **B** or initial states **D**) simply by replacing all **A**'s with **A**, **B**, **D**, and so on."

Given the lack of relevance to this paper specifically, and the more detailed unpacking elsewhere, we felt it would overburden the paper to include exhaustive equations for active inference (i.e., expressions that include a series of constants that play no role in the current application).

Equation 2: In the second equality, expected information gain formulations are used, and in the third equality, the risk and ambiguity formulation is used instead. However, the expected information gain about parameters seems to stay in the same form (except formulated as a KL divergence). Does the beliefs over parameters not have an ambiguity formulation, for example?

One could do so, but it is more complex to compute compared to the simple matrix operations to compute ambiguity from states. It is much simpler to compute the expected information gain formulation for parameters directly. We have kept it this way to be closer to the software implementation, but we fully agree there are many more permutations of terms that we could have presented here.

p.8: It might perhaps be useful to distinguish the predictive posterior from the posterior over states notationally somehow, such as with a tilde above the q, or similar.

We have used such notation in the past but feel it is clearer to specify the definition explicitly as we have done, and to make clear the support either from context or explicitly when used.

p.8. "Here, Q:= P…". a seems to be used instead of A here.

Thanks—we have now corrected this.

p.8. "The prior in (3)…" equation 3 seems to have multiple priors – perhaps it can be explicated which one is meant.

We now say:

> "The prior in **the first line of** (3) …"

Equation 5: mu is here used as a function of the Dirichlet parameters a and b. I presume this means taking the mean of the Dirichlet distribution to get the categorical distribution. If so, this might be made explicit.

Thanks—we have added:

> "Please see Figures 1 and 2 for definitions of the operators used here."

Equation 5: The authors might make clear that these equations are now implementations of the previous equations, and are specific to the discrete POMDP generative model they use in the paper, whereas the previous equations were more general.

Both implicitly commit to the same POMDP architecture as they depend upon specific conditional dependencies between the elements of this model.

Equation 5: this equation might be explained in a little more detail, similar to how the previous equations were explained.

Thanks, we have added:

> "This equation effectively gives specific implementational details—i.e., the way in which one can compute this quantity mathematically—of (3). Please see above for a conceptual interpretation."

Equation 7: While this equation is probably described and motivated in more detail in the article referred to, it might be nice to go a bit more in depth here, since it is central to the extension this paper presents. This includes making clear what the stylised B represents, and why the final equality is the case.

This is now made explicit as:

> "Using B to denote the beta function, we have (Friston, Parr et al. 2018) **the following expression for the log Bayes factor (or difference between log marginal likelihoods) between a full and reduced model**:"

p. 12 "entail a high mutual information". Why is it the case that there is a high mutual information between latent states and observations in the full set of models? Does the formalism not hold if some of the models being considered imply a low mutual information?

We did not fully understand this question. The passage in question motivates the need for models to include this high mutual information in terms of (3), which says that the prior over parameters scales with this mutual information.

Equation 10: why are m and A not used in the parameter and state information gain terms here? Are they marginalised, omitted for simplicity, or is it a consequence of the factorisation into model, parameter and state space?

This is just an application of the product rule of probability in logarithmic form: $\ln P(x,y,z) = \ln P(x|y,z) + \ln P(y|z) + \ln P(z)$.

Equation 11: the delta used as left hand side in the final equality is not explained anywhere; it would be useful to do so, I think.

We now say:

> "The Δ symbol is defined as in the final line, so that the first line can be expressed concisely."

p. 13 "note that this expected…" Why does the expected information gain not work unless parameters are updated over successive time points? And why are they not? Is this by the assumption of separated timescales, where beliefs over parameters are not updated on every time point, or for a different reason?

We have added:

> "Note that this expected information gain does not lend itself to evaluation over sequences of actions, unless the parameters are updated over successive time points in the future**, as the expected information gain over models is a function of the parameter updates, while parameter updates depend upon the states inferred over time.**"

p. 15. "preclude a learnable state-action policy" Why exactly is it not possible to use traditional reinforcement learning methods for sequential policy optimisation (or for partially observable

environments)? And is this also the case when using standard Bayesian learning methods, such as the standard parameter learning scheme in active inference discrete POMDPs?

The term 'state-action policy' is used here deliberately to answer this specific question. In principle, one could use versions of sequential policy optimisation that do not learn a mapping from all possible states to the actions to take from that state.

p. 16. "selected at random". Does this mean that the colour of the ball (i.e., the environment) depends on the agent's prior? Or does it refer to the agent assuming that the ball is sampled from D? And similarly, is there a difference between having a prior over the initial state (which may be fixed), and assuming that the initial state is sampled randomly? Here the second sounds like it is the case, but I would usually think of priors over latent states in the former way myself, so some clarity might be useful.

Recall that the only unknown amongst the priors is **A**. The **D** matches the environmental generative process. When one samples randomly, one does so from a distribution (fixed or otherwise)—in this case a prior. We did not fully understand the dichotomy you propose but would be happy to revisit this if you thought it important.

p. 16. The active inference model here selects its two actions (where to look and which colour to choose as its response) simultaneously. If a human were to do the three-ball task in an experimental setting, they would probably do them one at a time (i.e., first look, then decide, then look and so on). The authors might briefly comment on this, if nothing else just to make sure that the reader is not confused by expecting it to happen one at a time.

Please see Figure 3. Note the choice is delayed until a couple of eye movements have already taken place: exactly the sequence you describe.

p. 18. "Avogadro's number": the authors might briefly explain why this is relevant here

This was initially chosen as a reference big number of a comparable order of magnitude to the number of models. However, there was a calculation error initially in the number of models which makes this less relevant and we have removed this.

p. 18. "a true outcome": this is ambiguous – maybe write that they generate an outcome that the response was correct

Thanks—we have changed this to 'correct feedback'.

p. 21. "pose or colour": typo

Apologies, we could not identify the typo here. Perhaps the UK English spelling of colour?

Figure 4: the lowest two panels to the left have "trials" on the y-axis, while it is on the x-axis on all other panels in this figure. I would suggest changing this to keep it consistent and easier to read.

We appreciate your point here, but given there are fewer trials than there are latent variables, and we wish to align the latent variables across the true and inferred plots, this configuration is much more efficient in terms of the spatial layout of the figure.

Figure 4: the third panel, titled "divergence" seems to not be explained in the figure text. The divergence between what? It seems to be between the true and estimated parameter values.

Thanks for pointing this out. We have edited (in bold) the figure legend to say:

> "Following model selection, the posterior likelihoods converge on the true likelihoods and the divergence **(between the two)** in the third panel falls to 0."

There are often a lot of repetition between figure texts and the main text (such as for Figure 4). The authors can consider if this is necessary, or if some of the figure texts could be removed, in particular the parts that pertain more to the interpretation of the figures.

Thank you for raising this. We feel that it is important to include some interpretative detail in the figure legends for ease of reading these, but would be open to reconsidering this.

Figure 6: Would it be useful to do this analysis only with information gain about models, or only about models and parameters, or is there are principled reason to only use the four settings in the current plot?

As outlined above, the information gains at each level depend upon forming good inferences, supported by information gains, at the levels below. One could of course exhaustively examine every combination, but we felt the combinations assessed here speak more directly to the stepwise improvement in inference by adding information seeking behaviour at each level of inference and learning sequentially.

Figure 6: It might be nice to get a sense of the resulting parameter estimates (since these are arguably the point of the model reduction): do they get close to the true parameter values?

Please see Figure 4 lower left panels (yes, they do).

p. 26. "using the same initial conditions for each trial": Why repeat the analysis with the same initial conditions? It would be nice to see if the simulations are robust to different initial conditions or not. It seems the plots in figure 7 could also easily accommodate more data points.

This is to ensure the effects seen are not due to random effects in initial conditions and are attributable to the changes we have made between the conditions being assessed. We note you have omitted the word 'random' here, which might help to resolve this point. However, for greater clarity, we now say:

> "…using the same **sequence of** random initial conditions for each trial"

p. 27. "discovery time from": typo

Thanks—corrected.

Figure 7: since it is the primary focus on the article, there should arguably be a panel showing the effect of just removing expected information gain about models, without also removing the expected information gain about parameters. Otherwise, it is hard to judge whether the additional information gain indeed does have an advantage in this specific task.

Thank you—we have incorporated this. We have also taken the opportunity to make the plots a little prettier and more intuitive. The figure legend and accompanying text has been revised to include the additional comparisons:

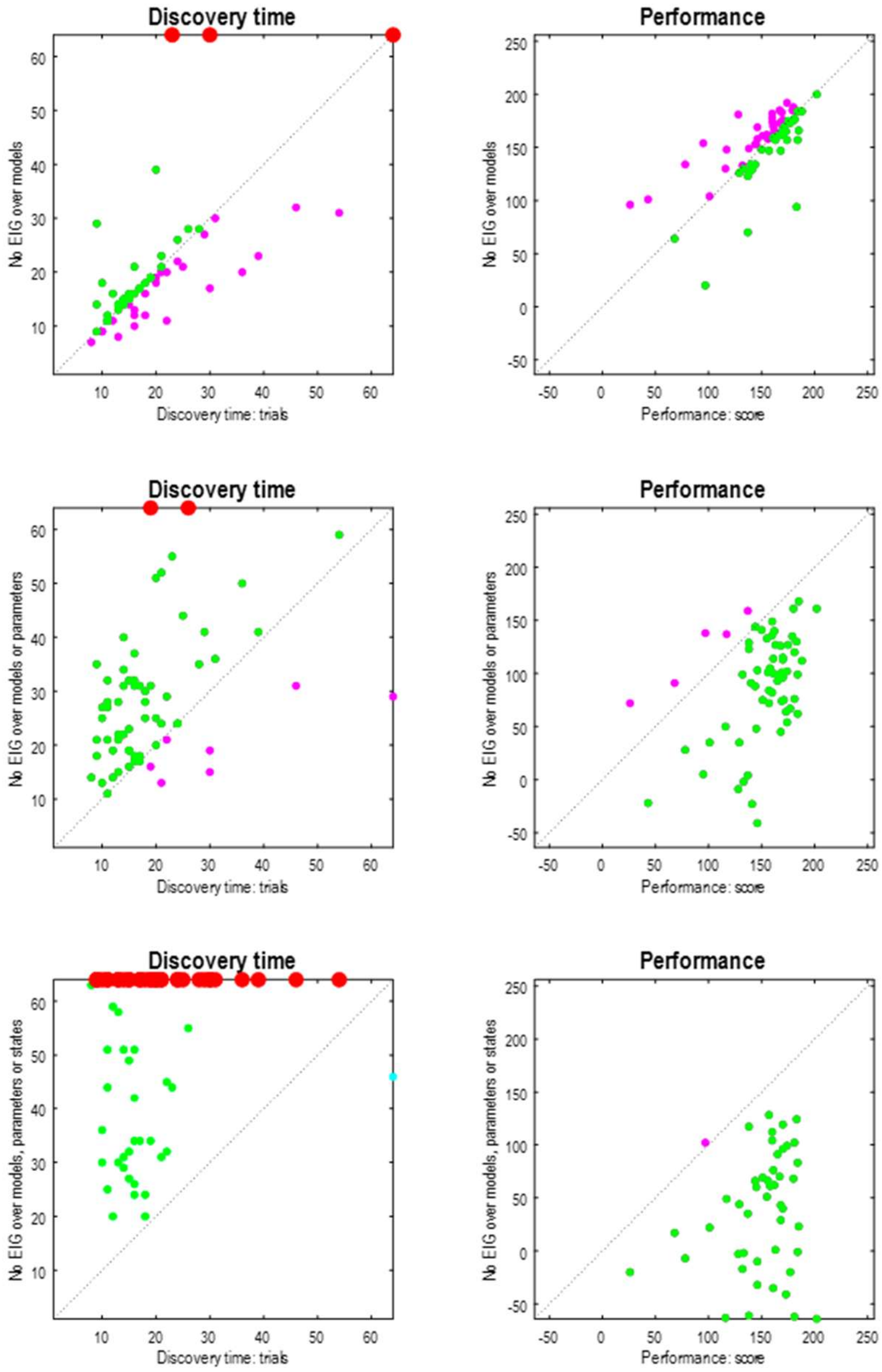

Figure 7 [now 8]: the importance of being curious. This figure shows the discovery time and performance when repeating the simulations in Figure 5 with and without expected information gain over models (upper row), models or parameters (middle row), and models, parameters or states (lower row). Each dot corresponds to a game with the same sequence of cues. The green dots report worse performance in the absence of expected information gain. A failure to discover the rule after 64 trials is arbitrarily assigned a discovery time of 64 (red dots). For example, the upper row shows that removing expected information gain over models precluded rule discovery in two of the 64 games (one game was not discovered in either case). These results illustrate the synergistic effect of expected information gain

over models, parameters and states, with almost universal improvement in rule discovery when all three are informing each other.

Figure 7: "same sequence of cues". Does this just mean that there are the same ball colours on each trial across simulations? Is this the same as having the same initial conditions? At first I thought the cues would be action-dependent, since they depend on where the agent looks – in this case, having the same cues across simulations would not be appropriate. This might be clarified by the authors, and, as I said, they might extend the simulation to different sets of cues to see how well their results generalise.

No, it means the same sequence across trials. Cues can be present whether the agent looks at them or not. We feel this is consistent with how cues would be thought of in (for example) experimental psychology. There are different cues between trials, although the sequence is consistent across them.

p. 28. "Here, partial observability…" Arguably, the most relevant unobservable variable here is the rule / criterion, rather than the color of the other balls.

Yes, entirely relevant.

p. 29. First paragraph: this description of the Bayesian model reduction approach would perhaps be nice to have earlier in the article, when the method is introduced.

We agree. We have moved the following part of this paragraph to the relevant methodological section:

> "The use of Bayesian model reduction in this setting casts each alternative model as a reduced version of the Bayesian model average under uniform priors. In other words, each model is defined by removing (combinations of) parameters through suitable shrinkage priors; c.f., (Efron and Morris 1973, Wipf and Rao 2007, Friston and Penny 2011). In the implementation adopted in this work, one simply applies shrinkage priors by setting Dirichlet counts to zero or a small value (e.g., 1/32). This provides a fast and efficient scheme for scoring the likelihood of different models, given just the original priors and a posterior that can be updated."

p. 29. "'what' and 'where": it seems here that the above formulation requires a factorisation of latent states into these two classes, but it is unclear whether this is a formal necessity for the kind of inference shown in this paper, or derived from the general statement that (human) brain have this factorisation. It seems to me that the model reduction and the calculation of the model space expected information gain do not specifically require the "what" and "where" factorisation.

There may be a misunderstanding here, which we hope the additional figure above helps to resolve. We have not suggested that factorisation is necessary for model reduction or information gain (it is not, as you rightly say). However, it is relevant for specification of a model space, as it is the relationships between factors that afford the symmetries used in constructing a plausible set of rules.

Remarks on code availability:

I have not reviewed the code underlying the simulation. It is included in the most recent release of the SPM software, although the authors have not specified where the file is or what it is called. I am happy to review the code, but would need to know which file refers to the simulations used in this article.

Thanks for offering to do so. We have now specified that it is within the DEM toolbox of SPM and is called DEM_reasoning.m. This is also available on GitHub: spm/toolbox/DEM/DEM_reasoning.m at main · spm/spm · GitHub.

We hope the above revisions and responses are what you had in mind.

Reviewer #3

The authors extend the active inference framework beyond state and parameter inference to structure learning via Bayesian model reduction, generalising the expected free energy objective over a predefined model space. An Occam's razor criterion applied to model posteriors yields a formal account of curiosity-driven inference and moments of realisation. The approach is validated on the three-ball paradigm, a rule-based visual search task, with empirical focus restricted to likelihood mapping discovery within a carefully constrained hypothesis space. Results show that the agent's posterior beliefs converge to a single most probable model when combined with the Occam's razor decision criterion.

Many thanks for this clear summary and for your helpful comments below.

That being said, I have the following major concerns:

1. It is not entirely clear whether the restricted model class constitutes a genuinely novel structure learning approach or a regularised parameter learning scheme. Broader notions of structure learning — such as hierarchical depth or latent dimensionality — are neither addressed nor discussed as limitations.

Thanks for highlighting this. We have added the following (new material in bold):

> "This means that the kind of reasoning demonstrated above rests upon learning a generative model of the world—and the consequences of acting upon that world—before rule learning.
>
> **Other advances that might help fill in the gaps here include broader perspectives on structure learning, ranging from generic schemes like hierarchical Dirichlet process modelling (Teh, Jordan et al. 2006, van Erp, Nuijten et al. 2025) through proposed neural implementations (Gershman and Niv 2010, Tenenbaum, Kemp et al. 2011) and applications of active inference approaches that learn the sorts of models we have used here (Smith, Schwartenbeck et al. 2020, Friston, Heins et al. 2024, Friston, Da Costa et al. 2024). The challenges one has to solve—when building a model of this sort—include determination of the appropriate hierarchical depth, the dimensionality of each state factor, the number of state factors, the number of plausible paths, and so on. These are challenging problems. However, in principle they can be addressed by making a series of changes to a model (either to grow or reduce it) and assessing the model evidence before and after each change. This, of course, raises further problems, which is the way in which one determines the sequence of changes to test and, if within one's control, the curriculum of observations on which to train a model. Solutions to these problems will be necessary to develop the models on which active reasoning rests.**
>
> **Once** the functional form and structure of the generative model have been learned…"

2. Empirical studies are conducted only for the likelihood mapping. Although learning the full model from scratch is discussed, it is not tested, leaving a significant gap between the theoretical claims and the empirical validation.

This is a fair comment. However, the theoretical claims made here specifically relate to the problem of rule learning which is most naturally framed in terms of the likelihood mapping. We hope that the following part of the discussion makes clear that we do not claim this form of active reasoning offers a complete account of structure learning from scratch:

> "… active reasoning, as described in this paper, only addresses a small part of this challenge. First, we have assumed that the agent knows the generative model under which the (unknown) rule is implemented; as opposed to having to learn the generative model from sampling some unsegmented sensory input, such as a pixel array. Furthermore, it has reduced the problem of rule discovery, or abstract reasoning, to discovering the form of a likelihood mapping to a rule-defining outcome. This means that the kind of reasoning demonstrated above rests upon learning a generative model of the world—and the consequences of acting upon that world—before rule learning."

If there are any points in the manuscript where you felt the theoretical claims implied more than what has been demonstrated, we would be more than happy to edit these.

3. The Bayesian model reduction framework is introduced without adequate explanation or intuition, making it difficult to follow even for a technically experienced reader.

Thanks for this. Based upon this, and advise from one of the other reviewers, we have moved a more intuitive description that originally appeared in the discussion earlier in the manuscript. The relevant text is:

> "The use of Bayesian model reduction in this setting casts each alternative model as a reduced version of the Bayesian model average under uniform priors. In other words, each model is defined by removing (combinations of) parameters through suitable shrinkage priors; c.f., (Efron and Morris 1973, Wipf and Rao 2007, Friston and Penny 2011). In the implementation adopted in this work, one simply applies shrinkage priors by setting Dirichlet counts to zero or a small value (e.g., 1/32). This provides a fast and efficient scheme for scoring the likelihood of different models, given just the original priors and a posterior that can be updated."

4. The construction of the restricted model class is dense and hard to follow; additional exposition or a worked example would substantially aid comprehension.

Thanks for raising this. We agree that a textual description here can be a little opaque and have now incorporated an additional figure (copied here for your convenience) in the hope that this offers greater intuitive transparency:

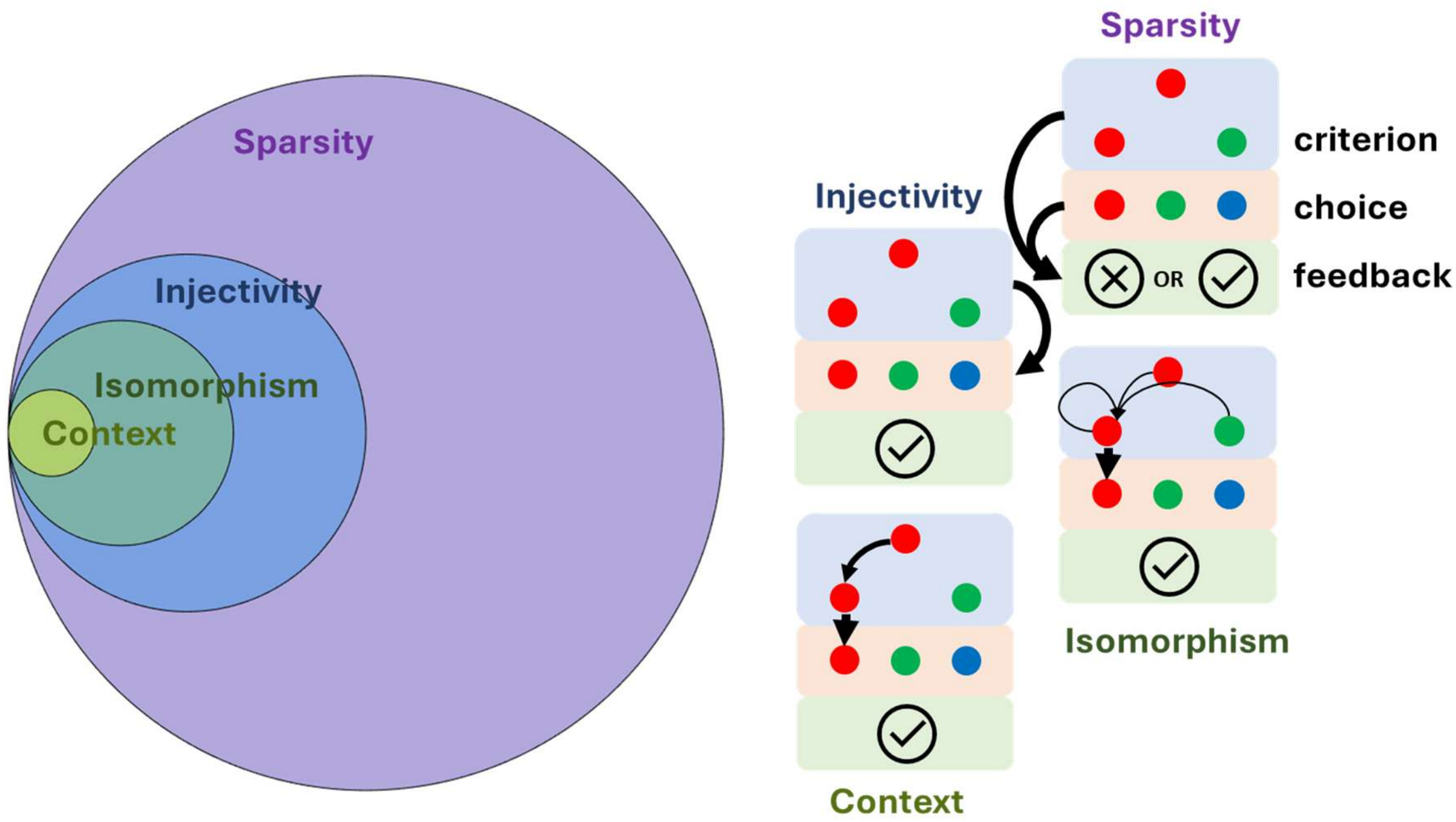

Figure 4: plausible rules. This figure is designed to give a graphical representation of the series of steps taken to limit the space of models used to those in which only plausible rules are in play. The graphic on the left shows the relative number of plausible rules (with the area of the circles scaling with the log number of rules) following application of each new constraint. The graphics on the right illustrate the kinds of rules included in each constraint. The initial sparsity constraint only allows for rules in which the feedback predicted is definitively positive or negative for any combination of choice and criterion states. The injectivity constraint says that, when the feedback is positive, there is an injective mapping from criterion to choice states. The isomorphism constraint says that one of the criterion factors that contains the same annotations (i.e., 'red', 'green', 'blue') as the choices. However, the specific factor (left, centre, or right cue) is here determined by some unknown context which may depend upon all three. Finally, the context constraint says that one of the criterion states (shown here as the centre state) provides all the contextual information required to determine the relevant criterion factor (shown here as the left state) that injectively and isomorphically maps to the choice state that ensures the feedback is positive. Please see the main text for more detail and pseudocode illustrating this.

Minor Concerns

• *Missing full stop at the end of: "First, it draws a clear distinction between the unknown variables entailed by a generative model; namely, latent states, parameters and structure, c.f., (Kemp and Tenenbaum, 2008)"*

Thanks—corrected.

• *Typographical error at the start of: "The So, does planning with epistemic intentions provide a sufficient account of reasoning?"*

Thanks—corrected.

Other questions for the authors

Introduction
• *How are the "a priori preferred" outcomes constructed?*

We have added:

> "These are specified in much the same way one might define value functions, with greater probability assigned to outcomes one expects to (behave to) achieve and low probability assigned to outcomes one expects to (behave to) avoid."

• *Can a time-, context-, and experience-dependent mapping from observations to expected free energy be learned?*

This is an interesting question. One answer might be that there is a well-defined functional mapping from a set of beliefs at a point in time to the expected free energy of the paths one could pursue from that point. This can only be learned from observations to the extent that the beliefs themselves are determined only by current observations. However, the premise of the information seeking element of expected free energy (in nontrivial settings) is that we expect beliefs to change with time, so that one needs the context of the last set of beliefs to be able to calculate the next. If one has all these things, one can just perform a direct calculation.

• *Please clarify the claim: "one cannot learn to be curious" — is this a theoretical limitation of the current framework or a broader claim?*

The original intention was in the spirit of our answer above. We have tried to address this technically (and heuristically) with the following footnote:

> "[1]Although it is possible to amortise (i.e., learn), time-independent mappings from observations to posteriors over states, one cannot learn a time-dependent mapping from a posterior over states and parameters to expected free energy. This is because the mapping changes with learning. Heuristically, one cannot learn to do something one has never done before. Having said this, it is perfectly possible to learn epistemic habits (Friston, FitzGerald et al. 2016); e.g., sitting down to watch the evening news. However, heuristically, one cannot learn to do something one has never done before."

If you think this is more confusing than helpful, we would be happy to remove the above claim.

Active Inference, Learning and Selection

• *How are the updates in the factor graph (Figure 2) derived, and what do the equality signs symbolise?*

They are derived from the fixed points of the variational free energy as per standard variational message passing:

> "The expressions in Figure 2 are effectively the fixed points (i.e., minima) of variational free energy. This means that message passing corresponds to a fixed-point iteration scheme (Beal 2003, Winn and Bishop 2005, Dauwels 2007)."

We have augmented the figure legend to say:

> "…where the nodes (square boxes) correspond to the factors of the generative model (labelled with the associated tensors). **Boxes containing the "=" symbol are Kronecker delta tensors that enforce equality between the connecting edges, ensuring the graph is bipartite.**"

• *Is there a missing subscript m on the calligraphic B in Equation 9?*

No, as the term inside the softmax function is the vector containing all elements of this, with the subscript m only relevant to indexing elements of the vector.

Three Ball Paradigm

• *How is the model space M constructed from different priors?*

Apologies, we should have specified this more clearly. We now say:

> "Each model within this space differs in that it contains different priors over the parameters of the distributions within the model. These imply different model structures in that some priors set some parameters as precisely zero (or close to), abolishing some conditional dependencies."

Conclusion / Scope

• *The authors position the work in relation to the ARC-AGI-3 challenge, but given the restrictive assumptions made — a known generative model structure, a predefined hypothesis space, and discrete state spaces — it is unclear whether and how the proposed approach could be applied to actual ARC-AGI-3 tasks. Could the authors elaborate on this?*

As we say, no, this work does not solve the whole problem to allow this application at this stage:

> "…active reasoning, as described in this paper, only addresses a small part of this challenge…"

However, if this is felt to be unclear, we would be happy to remove the mention of ARC-AGI-3 to avoid suggesting otherwise. Currently, we have used this to motivate the discussion you recommended about structure learning more broadly.

We hope the above revisions and responses are what you had in mind.

References

Albin, R. L., A. B. Young and J. B. Penney (1989). "The functional anatomy of basal ganglia disorders." Trends in neurosciences **12**(10): 366-375.

Beal, M. J. (2003). "Variational Algorithms for Approximate Bayesian Inference." PhD. Thesis, University College London.

Butz, M. and A. van Ooyen (2013). "A Simple Rule for Dendritic Spine and Axonal Bouton Formation Can Account for Cortical Reorganization after Focal Retinal Lesions." PLOS Computational Biology **9**(10): e1003259.

Cagnan, H., N. Mallet, C. K. E. Moll, A. Gulberti, A. B. Holt, M. Westphal, C. Gerloff, A. K. Engel, W. Hamel, P. J. Magill, P. Brown and A. Sharott (2019). "Temporal evolution of beta bursts in the parkinsonian cortical and basal ganglia network." Proceedings of the National Academy of Sciences **116**(32): 16095-16104.

Dauwels, J. (2007). On Variational Message Passing on Factor Graphs. 2007 IEEE International Symposium on Information Theory.

Deng, J. and A. Dunaevsky (2005). "Dynamics of dendritic spines and their afferent terminals: spines are more motile than presynaptic boutons." Developmental Biology **277**(2): 366-377.

Efron, B. and C. Morris (1973). "Stein's estimation rule and its competitors – an empirical Bayes approach." J Am Stats Assoc. **68**: 117-130.

Friston, K., L. Da Costa, D. Hafner, C. Hesp and T. Parr (2021). "Sophisticated Inference." Neural Comput **33**(3): 713-763.

Friston, K., L. Da Costa, D. A. R. Sakthivadivel, C. Heins, G. A. Pavliotis, M. Ramstead and T. Parr (2023). "Path integrals, particular kinds, and strange things." Physics of Life Reviews **47**: 35-62.

Friston, K., T. FitzGerald, F. Rigoli, P. Schwartenbeck, O. D. J and G. Pezzulo (2016). "Active inference and learning." Neurosci Biobehav Rev **68**: 862-879.
Friston, K., T. FitzGerald, F. Rigoli, P. Schwartenbeck and G. Pezzulo (2017). "Active Inference: A Process Theory." Neural Computation **29**(1): 1-49.
Friston, K., C. Heins, T. Verbelen, L. Da Costa, T. Salvatori, D. Markovic, A. Tschantz, M. Koudahl, C. Buckley and T. Parr (2024). "From pixels to planning: scale-free active inference." arXiv preprint arXiv:2407.20292.
Friston, K., C. Heins, T. Verbelen, L. Da Costa, T. Salvatori, D. Markovic, A. Tschantz, M. Koudahl, C. Buckley and T. Parr (2025). "From pixels to planning: scale-free active inference." Frontiers in Network Physiology **Volume 5 - 2025**.
Friston, K., T. Parr and P. Zeidman (2018). "Bayesian model reduction." arXiv preprint arXiv:1805.07092.
Friston, K. and W. Penny (2011). "Post hoc Bayesian model selection." NeuroImage **56**(4): 2089-2099.
Friston, K. and W. Penny (2011). "Post hoc Bayesian model selection." Neuroimage **56**(4): 2089-2099.
Friston, K., F. Rigoli, D. Ognibene, C. Mathys, T. Fitzgerald and G. Pezzulo (2015). "Active inference and epistemic value." Cognitive neuroscience(6:4): 187-214.
Friston, K. J., L. Da Costa, A. Tschantz, A. Kiefer, T. Salvatori, V. Neacsu, M. Koudahl, C. Heins, N. Sajid, D. Markovic, T. Parr, T. Verbelen and C. L. Buckley (2024). "Supervised structure learning." Biological Psychology **193**: 108891.
Friston, K. J., M. Lin, C. D. Frith, G. Pezzulo, J. A. Hobson and S. Ondobaka (2017). "Active Inference, Curiosity and Insight." Neural Comput **29**(10): 2633-2683.
Friston, K. J., V. Litvak, A. Oswal, A. Razi, K. E. Stephan, B. C. M. van Wijk, G. Ziegler and P. Zeidman (2016). "Bayesian model reduction and empirical Bayes for group (DCM) studies." NeuroImage **128**(Supplement C): 413-431.
Friston, K. J., T. Parr and B. de Vries (2017). "The graphical brain: Belief propagation and active inference." Network neuroscience (Cambridge, Mass.) **1**(4): 381-414.
Friston, K. J., R. Rosch, T. Parr, C. Price and H. Bowman (2017). "Deep temporal models and active inference." Neuroscience & Biobehavioral Reviews **77**: 388-402.
Gershman, S. J. and Y. Niv (2010). "Learning latent structure: carving nature at its joints." Curr Opin Neurobiol **20**(2): 251-256.
Henderson, P., J. Hu, J. Romoff, E. Brunskill, D. Jurafsky and J. Pineau (2020). "Towards the systematic reporting of the energy and carbon footprints of machine learning." The Journal of Machine Learning Research **21**(1): 10039-10081.
Hesp, C., R. Smith, T. Parr, M. Allen, K. J. Friston and M. J. D. Ramstead (2021). "Deeply Felt Affect: The Emergence of Valence in Deep Active Inference." Neural Computation **33**(2): 398-446.
Hinton, G. E., P. Dayan, B. J. Frey and R. M. Neal (1995). "The "wake-sleep" algorithm for unsupervised neural networks." Science **268**(5214): 1158-1161.
Koudahl, M. T., W. M. Kouw and B. de Vries (2021). "On Epistemics in Expected Free Energy for Linear Gaussian State Space Models." Entropy **23**(12): 1565.
Krawczyk, D. C. (2018). Chapter 3 - The Neuroscience of Reasoning. Reasoning, Academic Press**:** 41-69.
Marreiros, A. C., H. Cagnan, R. J. Moran, K. J. Friston and P. Brown (2013). "Basal ganglia–cortical interactions in Parkinsonian patients." NeuroImage **66**: 301-310.
Nambu, A. (2004). A new dynamic model of the cortico-basal ganglia loop. Progress in Brain Research, Elsevier. **Volume 143:** 461-466.
Parr, T., K. Friston and P. Zeidman (2024). "Active Data Selection and Information Seeking." Algorithms **17**(3): 118.
Parr, T. and K. J. Friston (2018). "The Anatomy of Inference: Generative Models and Brain Structure." Frontiers in Computational Neuroscience **12**(90).
Patterson, D., J. Gonzalez, Q. Le, C. Liang, L.-M. Munguia, D. Rothchild, D. So, M. Texier and J. Dean (2021). "Carbon emissions and large neural network training." arXiv preprint arXiv:2104.10350.
Prado, J., A. Chadha and J. R. Booth (2011). "The Brain Network for Deductive Reasoning: A Quantitative Meta-analysis of 28 Neuroimaging Studies." Journal of Cognitive Neuroscience **23**(11): 3483-3497.

Raven, J. C. (1941). "Standardization of progressive matrices, 1938." British Journal of Medical Psychology **19**: 137-150.
Rillig, M. C., M. Ågerstrand, M. Bi, K. A. Gould and U. Sauerland (2023). "Risks and Benefits of Large Language Models for the Environment." Environmental Science & Technology **57**(9): 3464-3466.
Shine, J. M., E. Matar, P. B. Ward, M. J. Frank, A. A. Moustafa, M. Pearson, S. L. Naismith and S. J. G. Lewis (2013). "Freezing of gait in Parkinson's disease is associated with functional decoupling between the cognitive control network and the basal ganglia." Brain **136**(12): 3671-3681.
Shipp, S. (2016). "Neural Elements for Predictive Coding." Frontiers in Psychology **7**: 1792.
Smith, R., P. Schwartenbeck, T. Parr and K. J. Friston (2020). "An Active Inference Approach to Modeling Structure Learning: Concept Learning as an Example Case." Frontiers in computational neuroscience **14**: 41-41.
Stettler, D. D., H. Yamahachi, W. Li, W. Denk and C. D. Gilbert (2006). "Axons and Synaptic Boutons Are Highly Dynamic in Adult Visual Cortex." Neuron **49**(6): 877-887.
Teh, Y. W., M. I. Jordan, M. J. Beal and D. M. Blei (2006). "Hierarchical Dirichlet Processes." Journal of the American Statistical Association **101**(476): 1566-1581.
Tenenbaum, J. B., C. Kemp, T. L. Griffiths and N. D. Goodman (2011). "How to grow a mind: statistics, structure, and abstraction." Science **331**(6022): 1279-1285.
van Erp, B., W. W. L. Nuijten and B. de Vries (2025). Online Structure Learning with Dirichlet Processes Through Message Passing, Cham, Springer Nature Switzerland.
Winn, J. and C. M. Bishop (2005). "Variational message passing." Journal of Machine Learning Research **6**: 661-694.
Wipf, D. P. and B. D. Rao (2007). "An empirical Bayesian strategy for solving the, simultaneous sparse approximation problem." Ieee Transactions on Signal Processing **55**(7): 3704-3716.

Review: Active inference and artificial reasoning

In this article, the authors propose an efficient method for extending the expected information gain component of the expected free energy, used as objective function for selecting action ins active inference, so that is also includes the expected information gain about model structure. They show in simulation studies that this method, which applies specifically when using Bayesian model reduction under fully discrete partially observed Markov decision process generative models, leads to increased sample efficiency as compared to only using information gain about states and parameters. The paper is well-written and consistent, and the introduced methodology is certainly relevant for the use of active inference models in the various contexts where they are used, including in artificial intelligence and for computational phenotyping such as in computational psychiatry. I am therefore happy to recommend this work for publication. I do, however, have a set of minor recommendations that I hope can contribute to improving the manuscript, such as regarding clarity, discussion of limitations, situating the work in the broader literature, and regarding the simulation methodology. Unless otherwise is mentioned, these should be seen as optional.

Clarity: technical extension or model of reasoning.

Throughout the article, it can feel, to me, somewhat unclear whether the aim of the work is to technically extend the active inference framework, or to provide a computational implementation of a theory of (human) reasoning. This should be made clear from the beginning.
If the aim is indeed to provide a model of reasoning, the paper should also include at least a brief overview of the literature already engaging with this goal, beyond the (often somewhat casual) use of the term in language models. It should also be justified why, exactly, being sensitive to information gain in model space when choosing where to look constitutes a model of reasoning. Finally, it would be useful to have at least a brief sketch in the discussion of how one might test this account of reasoning empirically in the future, such as by collecting behavioural data from reasoning tasks and investigating whether the augmented information gain term improves the ability of the model to capture the behaviour.
If the purpose is rather to technically extend the active inference framework to be sensitive to information gain in model space, and the term "reasoning" is used more casually here, the authors might make this more explicit early in the article.

Clarity: technical terminology

The article (and the active inference framework) uses a quite extensive technical terminology that is quite specific to its field, but also sometimes varies between articles and authors in it. It would be quite helpful to have an overview of this early

on, perhaps in a small box. Here I mean terms like “state”, “factor”, “observation”, “modality”, “path”, “policy”, “action”, “parameter”, “belief” and so on. This is especially relevant because different terms are used to describe the dimensionalities of states, observations and actions (i.e., factor, modality, and I am unsure what the value of a single timepoint for a single dimension of an action is called). This could also include the mathematical annotation for the different terms. Establishing such an overview might be nice for the active inference field in general.

The authors use “path” to refer to a single dimension of the action over time (distinguishing it from a “policy”, which refers to all the different action dimensions). In other fields, “paths” would not necessarily be considered one-dimensional, nor would they only refer to actions. Perhaps there is a better term than “path”? In any case, it is not clearly introduced before it is used in the figure text for Figure 1, which I expect would be confusing to many readers.

The authors early on use the term “learning” to refer specifically to parameter learning, and distinguish it from “selection”, which refers to model selection. However, they also call the model selection “rule learning”, and mention “learning a generative model”. To be consistent in their terminology, they might change “learning” to “parameter learning”, and use “learning” for parameter and model learning in general; or they might not use the term “learning” for the Bayesian model selection, despite the fact that this is often called “structure learning” in other fields. Finally, if the authors here intend for “structure learning”, “rule learning” and “learning a model” to be synonymous, they might make this explicit; if not, they might clarify the relations between these terms.

The authors call the kind of Bayesian model selection used in the paper a post-hoc approach. They might early on make clearly exactly what is meant with this. What makes it post-hoc?

Clarity: formal details

It might be useful if the digamma function and its role in the formalism is introduced briefly, to users who are not familiar with it.

If I understand correctly, a model-average of the various possible models is used in the beginning, until a single model is committed to. During this pre-commitment phase, parameter learning is done across all the possible models, which is feasible because it still only has to be done once (under this model reduction scheme) – the parameters can be reused, so to speak, because the different models are versions of the full model and the updated parameters are the same across models. Is that correct? Or does parameter learning not begin until the model has been selected? Since I was a bit in doubt, perhaps it is useful to clarify this in the article. Finally, it might be discussed whether there are formal limitations to this approach to Bayesian model reduction, at least if my first interpretation is correct. Is it an approximation, or

would it give the same results as if one used the (computationally heavy) approach of calculating the variational free energy for each sub-model separately and compared their model evidences?

Clarity: structure of introduction

The section titled “Active Inference, Learning and Selection” has some overlap with the introduction, and some of it would make reading the introduction easier. Section such as p. 3 starting with “Technically, variational free energy minus” would perhaps fit better in the “Active Inference, learning and selection” section, for example. The authors might consider if there is a way to streamline this first part of the article more.

Clarity: overview of contributions

The paper seems to make several, distinct, and increasingly specific, contributions. One is treating the choice of model structure as a random variable in its own right, and including the expected information gain for this variable in the expected free energy. Another is to apply this specifically for the already introduced case of Bayesian model reduction used here, where submodels of the full model can be specified by setting parameter values to 0. A third is to provide a manageable subset of models for the three-ball task (by imposing various sparsity constraints) which allows using the above method in practice. A final contribution is to provide an Ockam’s Razor approach where the agent moves from having a belief distribution about which model is true to committing to using only one model, only after which it has preferences about its observations. Giving a clear overview in this fashion early in the article might be useful for readers to understand where the contributions are, and how they depend on each other.

Limitations: specific generative model type

The authors use a somewhat specific type of generative model here, namely a discrete state-, observation-, action-, time- and model-, but continuous parameter-, space partially observable Markov decision process. Only laten states have dynamics and depend on their own previous state; observations only depend on states, and parameters have no dynamics at all. In addition, it is only transition models parameterised by B that depend on actions (and not the observation model parameterised by A, nor the model space). This model family is of course ubiquitous in the active inference as generative model, but it might still be useful to clarify briefly the limiting assumptions it makes in general. More specifically, it would be useful to discuss whether the methods introduced in this article also apply to other types of generative model, such as continuous state space models.

It might also be useful to clarify to which degree further limiting assumptions are imposed by this type of Bayesian model reduction and calculation of the model space expected information gain. In its current form, it seems to be incompatible with temporally deep planning. Is it also incompatible with hierarchically deep generative models? It does seem compatible with counterfactually deep generative models. Is it compatible with sophisticated inference or inductive inference? Would it interact in any relevant way with either of the above?

Limitations: only compatible with Bayesian model reduction

The authors might point out that the method they use is here is compatible only for use with the type of Bayesian model reduction they use (although not the general principle or including information gain about a random variable controlling the choice of model). This of course has advantages, as the authors mention, such as making the calculation highly efficient.
First, the authors might motivate this requirement for speed a little; is it computationally infeasible to use other methods, or is the speed primarily relevant for applications that are highly speed-sensitive?
In addition, the authors might consider to which degree this Bayesian model reduction approach is plausible as a model of (human) cognition. Is it likely that humans would separately evaluate a model evidence for a pre-specified set of submodels? 81 models still seem many, at least for it to be done consciously. Additionally, this method require the model space to be fully pre-specified. In which cases could this be expected?

Limitations: rule learning, not structure learning

In this article, the authors focus specifically on rule-learning, which is arguably a special case of the much broader task of structure learning. In this context, the authors use a series of constraints to *a priori* reduce the space of possible models. This includes (if I understand correctly) assuming that a) a set of latent states only can result in a single feedback outcome, b) that rules are always a combination of an action and a single criterion latent state, and c) that a criterion laten state is fully determined by a single latent context state. These assumptions are of course appropriate for this version of the three-ball paradigm, but do not apply even to slightly different versions of the task (such as if the correct answer depended on more than a single position). The authors should make these limitations explicit early on (they are stated, but are somewhat difficult to read). A visual depiction of how these constraints limit the space of possible models would be quite useful, I think. The authors might discuss to which degree their approach might generalise to other cases, and discuss to which degree this furnishes a generally applicable way to *a*

priori limit the model space. Finally, they might discuss whether a human participant is likely to approach this task with the same constraints or not.

Limitations: the three-ball paradigm

The authors might discuss the limitations imposed by using the three-ball paradigm in their simulations. It is for example perhaps a task where the information in model space is quite strongly dependent on the set of cues given to the agent, and arguably less dependent on the action the agent takes – in other words, I wonder if it is a task where the effect of including model space expected information gain is smaller than it might be in a task where action very strongly determine the effect of information one gets about model outcomes. The three-ball paradigm is also a task where, it seems, a main avenue for model space information is to make guesses given the cues a lot (as is seen in the simulation), which quite starkly impacts the pragmatic gain under non-flat preference priors (probably why flattening the preference priors is necessary in the beginning). One could easily imagine other tasks where it is possible to have preferences also during the model selection process.

Limitations: Occam's Razor to one model

The authors use what they call an Ockam's Razor approach, where the agent has an initial phase where it does Bayesian model comparison, until the Bayes factor reaches a threshold that triggers committing fully to a single model. Only after this does the agent have preferences for the observations it gets.

The authors should discuss this modelling in more detail, and discuss the alternatives.

First, what are alternatives? Arguably, a simpler approach is to just to use a Bayesian model average of the different models when planning, for example. If the posterior in model space is sharp, it seems this should become identical to the single-model approach.

Second, what are the justifications for the approach in the paper? Is it primarily the lessened computational load of committing to a single model? Is it informational, that averaging across models will lead to unidentifiability in state inference, for example? Or is it motivated by an account of (human) cognition, where people necessarily commit to a single hypothesis before acting on it? Or is it rooted in other principles from the Bayesian mechanics that active inference rely on ?

Third, what are the limitations of the method? It is for example not possible to change one's mind after committing to the model, even if strong evidence against it is provided afterwards.

Fourth, the authors might discuss the fact that they use a threshold for choosing the model (as opposed to a continuous belief change, which is often presented as an advantage of Bayesian methods over other approaches). The choice of threshold seems somewhat arbitrary; would the authors expect to later let this threshold depend on a component of the generative model(s), such as a cost of being wrong in model space? Is there a way to derive this threshold in a principled manner? Would the threshold be context-sensitive?

Finally, the authors might discuss and justify the choice of letting the agent have flat prior preferences before committing to a model.

Background: other kinds of structure learning

The Bayesian model reduction scheme used here is of course a special case of Bayesian model selection and structure learning in general. It could be useful if the authors situated their work somewhat within the larger field of Bayesian model selection, especially to discuss to which degree expected information gain could be calculated within other approaches. Would this approach for example be useful in the context of causal discovery, as a way to select informative actions about causal connections in a generative model (such as in Dynamical Causal Modelling)? Could it be used for model comparison where it is not the connections but different dynamics that are compared instead (such as is often done when modelling continuous state space phenomena). Is there a reason why priors in model space must be flat, beyond the use for the specific task here where we assume the agent has no previous knowledge about rules? Could it be combined with traditional methods that use (approximations of) the model evidence to select between models? Since a random variable controls the model, could this random variable have dynamics (i.e., a game where the rule changes, so that a different model becomes true at some point during the game)?

An obvious extension, which may seem closer to (human) reasoning, and which could benefit greatly from the sample-efficient information seeking behaviour provided by this article, would be in open-ended structure learning scenarios where a model is “grown” (i.e., where new causes are added and removed iteratively), such as in Dirichlet process models. The authors might briefly discuss whether and to which degree it would be possible to extend their method to include these use cases in the future.

Finally, there is also other work where Bayesian model selection is implemented by having a random variable control the model, such as in hierarchical mixture models. I know these best from fields using other approximations, such as sampling, to Bayesian inference, but they might still be worth mentioning in this context. Finally, the authors might discuss whether they would, in general, consider Bayesian model selection a special case of Bayesian inference over a latent variable that select

between different model structures, or if this is a specific approach used in this article that should be considered distinct from other kinds of Bayesian model comparison.

Simulation methodology:

The authors run several simulation studies to investigate the effect of adding the model space expected information gain to the expected free energy. They do not clearly show this effect, however, since they show the difference when removing expected information in both model and parameter space simultaneously. Since this is arguably the main result in the article, it should be shown clearly. Secondly, they run the simulation only using the same initial conditions, and with the same set of cues, which makes it difficult to know how well their results generalise. I would also strongly recommend addressing this.

See the last section with the specific comments for more information.

Other suggestions for discussion

The authors might consider briefly sketching out how the current formulation would interact with postdiction (i.e., re-inferring past states when receiving new information). As I understand, it is already implicit in the message passing scheme used that beliefs about a given state depend on knowledge about later states. Information gain in model space would supposedly also help disambiguating beliefs about past states.

The authors might discuss briefly whether this augmented version of the expected free energy is also derivable from the free energy principle. I expect it to be, since it seems to be just treating the model as another random variable to have beliefs about.

The authors might discuss how this kind of model reduction might be implemented neuronally; would it be a sudden change in connection strengths between neurons, or some other mechanism? How does it relate to proposed neurobiological implementations of the Dirichlet parameters, which the model reduction seems to act on?

Language, formatting and minor suggestions

p.1 “exploratory, information-seeking actions”: one could also add that “exploration” terms in many models are also essentially just added randomness or propensity to take actions not taken before, where it is here targeted specifically at sampling observations that are expected to be informative given current beliefs.

p.1 “homologue of “thinking”: the authors might justify this briefly, or at least indicate that it depends on what one means with “thinking”. Indeed, thinking is sometimes defined as language-based or explicit/aware, neither of which are *a priori* compatible with the planning as inference account.

p.2 “one cannot learn to be curious”: I would expect one could train a mapping from current beliefs and observations to the expected free energy – perhaps I am mistaken

p.2 “The So,”: typo

p.3 “cover abstraction”: the authors might clarify how exactly abstraction and reasoning are equated, and also why model space information gain sensitivity could be considered a type of abstraction.

p.3 “under the partially observed”: the authors might here and henceforth make clear that they refer specifically to discrete state, action and observation POMDPs, since the following formalism doesn’t seem to apply to continuous formulations. They do mention this at times, but it might be nice to clear from the beginning.

Figure 1. “empirical prior”: This use of the term seems to diverge from how it is used in Bayesian inference to describe priors chosen based on previous work and data. Since “empirical” seems to denote something dependent on empirical data, it seems somewhat odd to use it for priors that depend on other variables in the model. Perhaps there is a standard term for this in the broader Bayesian inference world? I suppose this is often what the term “hyperprior” targets, although this of course describe the random variables which the “empirical priors” depend on.

Figure 1. The optimisations in the bottom-right corner of the figure are not explained in much detail in the figure text, and only some of it in the main text quite a bit later (including the cursive l function of the E matrix, or the difference between the bold and non-bold matrix names). Perhaps giving a brief explanation here would be useful.

Equation 1: Should there be a subscript T for the variational free energy F on the left side? I suppose this will be the F at a specific point in time, and the right hand side of the equations uses t subscripts.

Equation 1: the dependence on the model *m* is explicated for the log model evidence p(o|m). I suppose the dependence on the model is also implicit in many of the other terms, however. The authors might consider making this consistent, and if not, stating clearly that omit the model dependency only in some terms. This is particularly relevant because this paper focuses specifically on model structure learning.

Equation 1: when calculating the prior in the first line, the transition from s-1 to 1 is calculated relative to the action u at time t (not t-1). Perhaps I am mistaken, but I

would expect that it is the action at the previous timestep that controls this transition, since the action at the current timestep hasn't happened yet.

Equation 1: In the final line, in the term for calculating the complexity, I suppose the true posterior over states, actions and parameters will also be conditional on the previous action, and not just the previous state?

Equation 2: The authors point out that they omit some parts of the full equation here. This is fine, but of course can make the mathematics more opaque. I would find it useful if they would include a fully spelled out version of this and other equations in the appendix.

Equation 2: In the second equality, expected information gain formulations are used, and in the third equality, the risk and ambiguity formulation is used instead. However, the expected information gain about parameters seems to stay in the same form (except formulated as a KL divergence). Does the beliefs over parameters not have an ambiguity formulation, for example?

p.8: It might perhaps be useful to distinguish the predictive posterior from the posterior over states notationally somehow, such as with a tilde above the q, or similar.

p.8. "Here, Q:= P…". *a* seems to be used instead of A here.

p.8. "The prior in (3)…" equation 3 seems to have multiple priors – perhaps it can be explicated which one is meant.

Equation 5: *mu* is here used as a function of the Dirichlet parameters a and b. I presume this means taking the mean of the Dirichlet distribution to get the categorical distribution. If so, this might be made explicit.

Equation 5: The authors might make clear that these equations are now implementations of the previous equations, and are specific to the discrete POMDP generative model they use in the paper, whereas the previous equations were more general.

Equation 5: this equation might be explained in a little more detail, similar to how the previous equations were explained.

Equation 7: While this equation is probably described and motivated in more detail in the article referred to, it might be nice to go a bit more in depth here, since it is central to the extension this paper presents. This includes making clear what the stylised B represents, and why the final equality is the case.

p. 12 "entail a high mutual information". Why is it the case that there is a high mutual information between latent states and observations in the full set of models? Does the formalism not hold if some of the models being considered imply a low mutual information?

Equation 10: why are m and A not used in the parameter and state information gain terms here? Are they marginalised, omitted for simplicity, or is it a consequence of the factorisation into model, parameter and state space?

Equation 11: the delta used as left hand side in the final equality is not explained anywhere; it would be useful to do so, I think.

p. 13 “note that this expected…” Why does the expected information gain not work unless parameters are updated over successive time points? And why are they not? Is this by the assumption of separated timescales, where beliefs over parameters are not updated on every time point, or for a different reason?

p. 15. “preclude a learnable state-action policy” Why exactly is it not possible to use traditional reinforcement learning methods for sequential policy optimisation (or for partially observable environments)? And is this also the case when using standard Bayesian learning methods, such as the standard parameter learning scheme in active inference discrete POMDPs?

p. 16. “selected at random”. Does this mean that the colour of the ball (i.e., the environment) depends on the agent’s prior? Or does it refer to the agent assuming that the ball is sampled from D? And similarly, is there a difference between having a prior over the initial state (which may be fixed), and assuming that the initial state is sampled randomly? Here the second sounds like it is the case, but I would usually think of priors over latent states in the former way myself, so some clarity might be useful.

p. 16. The active inference model here selects its two actions (where to look and which colour to choose as its response) simultaneously. If a human were to do the three-ball task in an experimental setting, they would probably do them one at a time (i.e., first look, then decide, then look and so on). The authors might briefly comment on this, if nothing else just to make sure that the reader is not confused by expecting it to happen one at a time.

p. 18. “Avogadro’s number”: the authors might briefly explain why this is relevant here

p. 18. “a true outcome”: this is ambiguous – maybe write that they generate an outcome that the response was correct

p. 21. “pose or colour”: typo

Figure 4: the lowest two panels to the left have “trials” on the y-axis, while it is on the x-axis on all other panels in this figure. I would suggest changing this to keep it consistent and easier to read.

Figure 4: the third panel, titled “divergence” seems to not be explained in the figure text. The divergence between what? It seems to be between the true and estimated parameter values.

There are often a lot of repetition between figure texts and the main text (such as for Figure 4). The authors can consider if this is necessary, or if some of the figure texts could be removed, in particular the parts that pertain more to the interpretation of the figures.

Figure 6: Would it be useful to do this analysis only with information gain about models, or only about models and parameters, or is there are principled reason to only use the four settings in the current plot?

Figure 6: It might be nice to get a sense of the resulting parameter estimates (since these are arguably the point of the model reduction): do they get close to the true parameter values?

p. 26. “using the same initial conditions for each trial”: Why repeat the analysis with the same initial conditions? It would be nice to see if the simulations are robust to different initial conditions or not. It seems the plots in figure 7 could also easily accommodate more data points.

p. 27. “discovery time from”: typo

Figure 7: since it is the primary focus on the article, there should arguably be a panel showing the effect of just removing expected information gain about models, without also removing the expected information gain about parameters. Otherwise, it is hard to judge whether the additional information gain indeed does have an advantage in this specific task.

Figure 7: “same sequence of cues”. Does this just mean that there are the same ball colours on each trial across simulations? Is this the same as having the same initial conditions? At first I thought the cues would be action-dependent, since they depend on where the agent looks – in this case, having the same cues across simulations would not be appropriate. This might be clarified by the authors, and, as I said, they might extend the simulation to different sets of cues to see how well their results generalise.

p. 28. “Here, partial observability…” Arguably, the most relevant unobservable variable here is the rule / criterion, rather than the color of the other balls.

p. 29. First paragraph: this description of the Bayesian model reduction approach would perhaps be nice to have earlier in the article, when the method is introduced.

p. 29. “’what’ and ‘where”: it seems here that the above formulation requires a factorisation of latent states into these two classes, but it is unclear whether this is a formal necessity for the kind of inference shown in this paper, or derived from the general statement that (human) brain have this factorisation. It seems to me that the model reduction and the calculation of the model space expected information gain do not specifically require the “what” and “where” factorisation.
